# Multi-omics Characterization of Acquired Olaparib Resistance in BRCA1 and BRCA2 Mutant Breast Cancer Cell Lines

**DOI:** 10.1016/j.mcpro.2025.101034

**Published:** 2025-07-14

**Authors:** Holda A. Anagho-Mattanovich, Meeli Mullari, Matthias Anagho-Mattanovich, Hayoung Cho, Anna-Kathrine Pedersen, Oana Palasca, Jesper V. Olsen, Marie Locard-Paulet, Michael L. Nielsen

**Affiliations:** 1 Department of Proteomics, Novo Nordisk Foundation Center for Protein Research, Institute for Cellular and Molecular Biology, Faculty of Health and Medical Sciences, University of Copenhagen, Copenhagen, Denmark; 2 Novo Nordisk Foundation Center for Basic Metabolic Research, Faculty of Health and Medical Sciences, University of Copenhagen, Copenhagen, Denmark; 3 Institut de Pharmacologie et de Biologie Structurale (IPBS), Université de Toulouse, CNRS, Université Toulouse III - Paul Sabatier (UT3), Toulouse, France

**Keywords:** LC-MSMS, Olaparib, PARP inhibitors, BRCA1/2 mutant, Olaparib resistance, ADP-ribosylation, EThcD, proteomics, phosphoproteomics, DNA damage

## Abstract

Poly (ADP-ribose) polymerase inhibitors (PARPi) are widely used as targeted therapies against breast cancers with BRCA mutations. However, the development of resistance to PARPi poses a significant challenge for long-term efficacy of these therapies, warranting further understanding of mechanisms of PARPi resistance. Here, we generated and characterized Olaparib resistance in BRCA1/2 mutant breast cancer cell lines MDAMB436 and HCC1428 using a systems-level multi-omics approach, including transcriptome, proteome, phosphoproteome, and ADP-ribosylation analysis. Our analyses revealed that resistance development strongly correlated with protein expression changes, while modest effects on phosphorylation- and ADP-ribosylation-dependent signaling pathways were observed. We found that BRCA1 expression was reestablished in OR MDAMB436 cell lines, while PARP1 expression was decreased. In OR HCC1428 cell lines, the BRCA2 mutation was not reverted. However, we observed increased expression of Fanconi anemia group D2 (FANCD2), histone parylation factor 1 (HPF1), and Nicotinamide phosphoribosyltransferase (NAMPT) in various cell lines, suggesting increased replication fork protection, and changes in the ADPr pathway and adaptation of metabolic pathways as resistance mechanisms. Our findings provide valuable insights into the complex landscape of PARPi resistance, offering potential targets for further investigation and therapeutic intervention.

## Highlights

• System-wide analysis of ADP-ribosylation sites across Olaparib-resistant (OR) cells.
• Consistent ADP-ribosylation response across sensitive and resistant cells.
• Resistance development was strongly correlated with protein expression changes.
• BRCA1 expression is reestablished in OR MDAMB436 cell lines, while PARP1 expression is decreased.

## Graphical Abstract

**Figure:**
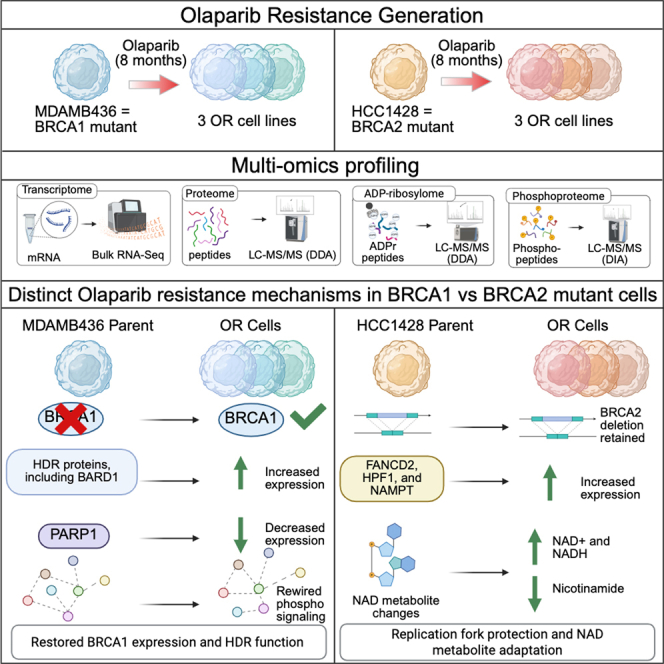

## In Brief

This study presents a multi-omics analysis of acquired Olaparib resistance in BRCA1- and BRCA2-mutant breast cancer cell lines. Analysis of the transcriptome, proteome, phosphoproteome, and ADP-ribosylome data revealed distinct resistance mechanisms: BRCA1 restoration and phosphoproteome-signaling rewiring in BRCA1-mutant cells and replication fork protection and metabolic adaptation in BRCA2-mutant cells.

BRCA1 and BRCA2 proteins are required for homology-directed repair (HDR) of DNA double-strand breaks (DSBs) (1, 2), and germline mutations in *BRCA1* or *BRCA2* genes predispose individuals to breast, ovarian, and other cancers (3). Tumors arising from cells with defects in HDR are characterized by severe chromosomal instability resulting from alternative error-prone forms of DNA repair (4). Notably, cells with loss-of-function mutations in BRCA1 or BRCA2 exhibit enhanced sensitivity to PARP inhibitors (PARPi) (5, 6). PARPi inhibits the catalytic function of ADP-ribosyltransferases, also known as Poly ADP-ribose polymerases (PARPs), enzymes that catalyze the transfer of ADP-ribose (ADPr) to target proteins using NAD+ as a substrate. The most abundant and active PARP, PARP1, is crucial for DNA damage repair as it binds to damaged DNA and ADP-ribosylates target proteins involved in various DNA damage repair processes (7, 8, 9). When cells are exposed to DNA damage, the histone PARylation factor (HPF1) associates with PARP1 or PARP2, the latter being an isoform that shares redundant functions with PARP1 (10). This interaction completes their active sites and directs ADP-ribosylation to serine residues (11, 12, 13, 14, 15).

The synthetic lethal interaction between PARP inhibition and BRCA1/2 mutations formed the basis for the approval of PARP inhibitors, such as Olaparib and Talazoparib, as monotherapies for patients with loss-of-function germline BRCA-mutated HER2-negative breast cancer (16). Clinical trials are also underway for patients with other HDR defects, aiming to expand the pool of patients who may benefit from these drugs. Patients treated with PARPi Olaparib or Talazoparib have had significantly increased progression-free survival times compared to patients on chemotherapy (17, 18). However, prolonged use of monotherapies inevitably leads to PARPi resistance, which is a major obstacle to treating patients with cancer.

The most common mechanism of PARPi resistance development in cells is through mutations that restore the function of BRCA1, BRCA2, or other proteins involved in HDR (19, 20, 21, 22, 23, 24). Cells can also gain PARPi resistance by reducing the bioavailability of the drug through upregulation of drug efflux transporter genes (25, 26), by decreased PARP trapping through disruption of PARP1 or the Poly(ADP-ribose) glycohydrolase (PARG) levels or activity (27, 28, 29, 30), or by increased protection of the replication fork through upregulation of the Fanconi anemia group D2 protein (FANCD2) (31). Understanding Olaparib resistance mechanisms has also provided insights into vulnerabilities that may be targeted to overcome PARPi resistance in the clinic. While PARPi resistance mechanisms have been a subject of extensive study, few have focused on a systems-level look at ADPr signaling within the context of acquired PARPi resistance. Therefore, our understanding may be further improved by characterization of the roles that ADPr signaling in DNA damage plays in this process.

With this in mind, we undertook a systems-level characterization of acquired Olaparib resistance in MDAMB436 and HCC1428 breast cancer cells, which carry BRCA1 and BRCA2 mutations, respectively. We first treated MDAMB436 and HCC1428 continuously with increasing concentrations of PARPi until they developed resistance to Olaparib. Using RNA sequencing and quantitative LC-MS/MS, we then characterized transcriptomes, proteomes, ADP-ribosylomes, and phosphoproteomes of untreated cells and cells treated with H_2_O_2_ to induce DNA damage ADP-ribosylation (ADPr) or pretreated with Olaparib before H_2_O_2_ treatment to inhibit PARP activity in the sensitive and OR cell lines.

We found that BRCA1 expression was restored in OR MDAMB436 cells, replicating the most frequently observed PARPi resistance mechanism in BRCA1 mutants (32). Basal DNA damage levels were also decreased in OR MDAMB436 cells, likely due to restored HDR capacity. Furthermore, there was a significant decrease in PARP1 expression, resulting in a modest reduction of the overall DNA damage ADPr signal in OR MDAMB436 cells. On the other hand, reversion of the BRCA2 mutation was not observed in OR HCC1428 cells. Instead, FANCD2 protein levels were significantly increased, suggesting replication fork protection as a likely resistance mechanism. Moreover, expression of the PARP1 cofactor HPF1 was also increased in OR HCC1428 cells, leading to a modest increase in H_2_O_2_-induced ADP-ribosylation. Expression of NAMPT, the rate-limiting enzyme for NAD+ production via the salvage pathway responsible for most of the NAD+ production in mammalian cells (33), was significantly increased at the RNA level in all HCC1428 OR cells, and was significantly up-regulated on the protein level in OR Cl A. This resulted in significantly lower nicotinamide levels and higher NAD+ and NADH levels compared to parent cells. Importantly, changes in ADPr signaling enzymes in MDAMB436 and HCC1428 cells corresponded with only modest DNA damage ADP-ribosylation differences in the cell lines, underscoring the robustness and homogeneity of DNA damage-induced ADPr signaling. Our data suggest that OR cells may use partially opposing strategies to develop resistance, including adapting to altered requirements for NAD+.

## Experimental Procedures

### Cell Lines

Immortalized breast cancer cell lines MDAMB436 and HCC1428, originally from the American Type Culture Collection, were kindly provided by Jesper Olsen (NNF-CPR, University of Copenhagen). Cells were grown in DMEM (Invitrogen) supplemented with 10% fetal bovine serum and penicillin/streptomycin (100 U/ml; Gibco) (cell culture medium) in a 37 °C incubator with 5% CO_2._

### Generation of Olaparib Resistance in MDAMB436 and HCC1428 cells

MDAMB436 and HCC1428 cells were cultured in 6-well plates with continuous exposure to increasing amounts of Olaparib (AZD2281 Ku-0059436, reconstituted in DMSO), with a starting concentration approximately corresponding to CC_50_ values of 5 nM for MDAMB436 cells and 0.2 μM for HCC1428 cells. CC_50_ values were determined previously using colony formation assays. Cells were passaged when confluent and Olaparib concentrations increased approximately every 2 weeks to a final concentration of 0.2 μM in MDAMB436 cells and 2 μM in HCC1428 cells after 8 months. Cells were then grown for 3 weeks without Olaparib before expanding cell lines for validation and further characterization. Colony formation assays were then performed to validate increased sensitivity to Olaparib. When not in culture, cells were stored in fetal bovine serum with 5% DMSO in a liquid nitrogen freezer. Three independent Olaparib-resistant (OR) cell lines were generated from MDAMB436 parent cells (A1, A2, and A3) and three from HCC1428 cells (A, B, and C). Visual checks in a light microscope showed that the OR cells retained a similar morphology to their respective parent cell lines. All OR and parent cells were routinely tested for *mycoplasma* contamination.

### Colony Formation Assays

MDAMB436 and HCC1428 parent cells and their respective OR cell lines were seeded evenly at 1000 cells/well in 6-well plates and incubated overnight. Cells were then treated with 0.001852, 0.011111, 0.066667, 0.4, 2, 5, or 10 μM Olaparib diluted in cell culture medium with a final DMSO concentration of 0.1%, and cell culture medium containing 1% DMSO was added to control wells. Each treatment was done in triplicate. Cells were cultured continuously in Olaparib for 15 days (MDAMB436 cells) or 19 days (HCC1428 cells), and Olaparib-containing media were refreshed every 3 to 4 days. Cells were fixed and stained with 0.5% Crystal violet solution (0.5% Crystal violet with 20% Methanol).

### Western Blotting

Cells grown to 80% confluency were washed twice with PBS, collected by gentle scraping with a cell scraper and centrifuged at 400×*g* for 3 min at room temperature. To assess ADPr signaling, cells were treated with 1 mM H_2_O_2_ in PBS for 10 min or pretreated with 10 μM Olaparib for 1 h before H_2_O_2_ treatment. The respective controls were treated with PBS or DMSO instead. After incubation at 37 °C, cells were collected and pelleted. Cell pellets were lysed with SDS lysis buffer (50 mM Tris pH 8.5, 150 mM NaCl, and 2% SDS) and incubated at 90 °C for 1 h on a benchtop heating block with shaking at 1400 rpm. Protein concentrations were determined by BCA assay (Thermo Fisher) following the manufacturer’s protocol. NuPAGE LDS sample buffer (Invitrogen) was added to equal amounts of cell lysates at a final concentration of 1×, after which lysates were boiled at 95 °C for 5 min. Lysates were separated by SDS-PAGE gel electrophoresis on 1 mM NuPAGE 4 to 12% bis-tris gels (Thermo Fisher) with MOPS running buffer. Separated proteins were transferred onto nitrocellulose membranes using Tris-Glycine-methanol transfer buffer (30.3 g Tris Base, 144g Glycine, and 20% Methanol in 1000 ml). Overall protein amounts were visually assessed with Ponceau staining, and the membranes were then washed for 10 min in PBS/0.1% Tween-20 (PBS-T). Membranes were blocked for 1 h in 5% BSA or 5% milk in PBS-T, depending on the primary antibody used. Membranes were incubated with primary antibodies overnight at 4 °C, washed three times with PBS-T, incubated with HRP-conjugated secondary goat antibodies to mouse or rabbit diluted 1:7500 in PBS-T with 5% milk (Rockland) for 1 h at room temperature, and washed three times with PBS-T. Proteins were detected on Amersham Hyperfilm ECL films (Sigma) using the Novex ECL Chemiluminescent Substrate Reagent Kit (Invitrogen). The following antibodies were used in this study: PARP1/2 Rabbit pAB sc-7150 (Santa Cruz Biotech) 1:200, PARG (D4E6X) Rabbit mAb #66564 (Cell Signaling), HPF1 Rabbit pAb HPA043467 (Atlas Antibodies), ARH3/ADPRHL2 Rabbit pAb HPA027104 (Atlas Antibodies), Poly/Mono-ADP Ribose (E6F6A) Rabbit mAb #83732 (Cell Signaling), and gamma-Tubulin Ms mAb T5326 (Sigma Aldrich) 1:2500. PARP1, PARG, HPF1, ARH3, and gamma-Tubulin antibodies were diluted in PBST with 5% BSA, and Poly/Mono-ADP Ribose antibody was diluted in PBST with 5% milk. Antibodies were used at a dilution of 1:1000 unless otherwise indicated.

### Experimental Design and Statistical Rationale for Omics Experiments

For the transcriptome, proteome, ADP-ribosylome, and phosphoproteome experiments, we analyzed four biological replicates per condition. After cell lysis (Mass Spectrometry experiments) or RNA extraction (transcriptomics), all replicates of the same condition were prepared together to minimize variability introduced during sample preparation. Single injections were performed, meaning no technical replicates were included. After data processing, proteins or sites identified in fewer than three replicates of a given condition were filtered out. The statistical tests employed for data analysis in this study require at least three replicates. Including four replicates and the filtering steps, therefore, captures the variability necessary for statistical inference.

### RNA Sequencing

Parent and OR MDAMB436 and HCC1428 cells were seeded in 6-well plates in DMEM (10% FBS/1% pen/strep) at 2 million cells per well. The next day, cells were washed with PBS, followed by lysis and RNA extraction using the Qiagen RNeasy kit (Qiagen) with the manufacturer’s instructions. RNA was quantified using a Nanodrop spectrophotometer (Thermo Scientific). After confirming RNA integrity using the TapeStation RNA ScreenTape (Agilent), mRNA was enriched using the NEBNext Poly(A) mRNA Magnetic Isolation Module (NEB-E7490; New England Biolabs). mRNA was used for cDNA synthesis and library preparation using the NEBNext Ultra II Directional RNA Library Prep (NEB-E7765; New England Biolabs) with NEBNext Multiplex Oligos for Illumina Set 1 (E6440) for indexing. The quality of sequencing libraries was confirmed with TapeStation High Sensitivity D1000 ScreenTape DNA kit (Agilent). Libraries were then diluted, pooled, and sequenced on NextSeq2000 with a P3 kit (50 cycles, single end, read 1 = 72 base pairs, index1 and 2 = 8 base pairs each).

### Bioinformatics Analysis of RNA Sequencing Data

Fastq files were generated using bcl2fastq v2.19.1 and aligned to the [hg38/GRCh38] genome using STAR v2.5.3a (34). Transcript expression levels were estimated with the quantMode GeneCounts option and [GRCh38p10.v27] annotations. FastQC v0.11.7 (http://www.bioinformatics.babraham.ac.uk/projects/fastqc) was used for QC metrics, and MultiQC v1.7 (35) for reporting. Data analysis was then performed using R/Bioconductor (https://www.R-project.org, (36)). Normalization and differential expression analysis were performed with DEseq2 v1.24.0 (37).

### Variant Calling on the RNA-Sequencing data

Variant calling analysis was conducted using a pipeline based on GATK v4.4 (38)^,^ following the best practices for RNA-Seq data. Fastq files were aligned to the hg38/GRCh38 genome using STAR v2.7.10a in two-pass mode. During the second mapping round, the genome index was built using splice junctions identified across all samples in the first mapping. Subsequently, duplicates were marked using Picard-tools v3.1 (https://broadinstitute.github.io/picard/). We applied SplitNCigarReads to prepare the data for variant calling, followed by two rounds of base recalibration. Variant calling was performed using GATK's Mutect2 in tumor-only mode, with the recommended germline resource and panel of normals reference files. Variants were filtered using FilterMutectCalls with default options and annotated with Annovar (39). To ensure high-confidence variants, we filtered results to require a minimum allelic depth of two reads for mutated alleles. To identify mutations acquired in resistant clones compared to parent cells, we selected variants present in at least three replicates of a clone and absent in all replicates of the parent cell line. Visualization of the filtered variants was performed using the R package maftools v2.18.0 (40).

### Proteome and ADP-Ribosylome Mass Spectrometry

#### Proteome MS Sample Preparation

Parent and OR cells were prepared for proteome mass spectrometry using standard shotgun proteomics sample preparation methods. Briefly, cells were grown to 80% confluency, washed twice with PBS, pelleted, and lysed with denaturing guanidinium-HCl lysis buffer (6M guanidine-HCl (G3272; Sigma Aldrich), 50 mM Tris pH 8.5) supplemented with 5 mM tris(2-carboxyethyl)phosphine (TCEP; C4706, Sigma Aldrich) and 10 mM chloroacetamide (CAA; 22790, Sigma Aldrich). Lysates were sonicated on ice for 45 s at 60% amplitude. After 1 h at room temperature, Lys-C endopeptidase (1:200 w/w; Wako Chemicals) was added to lysates for 3 h, followed by overnight incubation with modified sequencing-grade Trypsin (1:200 w/w; Sigma Aldrich) after diluting to 1.5 M GndHCl with 25 mM Tris pH 8.5. Trifluoroacetic acid (TFA) was then added to the sample at a final concentration of 0.5% v/v, and samples were centrifuged at high speed to remove precipitates. Peptides were purified using reverse-phase Sep-Pak C18 cartridges (WAT051910; Waters) according to the manufacturer’s protocol, eluted in 30% Acetonitrile (ACN) in 0.1% TFA, and vacuum-dried in a SpeedVac (Eppendorf) at 60 °C. Peptides were reconstituted in 50 mM Ammonium bicarbonate (ABC) and separated on an Acuity UPLC Peptide CSH C18 1.7 μm reversed-phase column (186006935; Waters) under basic conditions. Peptides were collected in 46 timed intervals and concatenated into 12 fractions (Batth and Olsen, 2016). Formic acid (FA) was added to peptides (0.5% v/v), and peptides were vacuum-dried at 60 °C and reconstituted in 0.1% FA for MS analysis.

#### ADPr MS Sample Preparation

Parent and OR cells grown to 80% confluency were treated with 1 mM H_2_O_2_ in PBS for 10 min or pre-treated with 10 μM Olaparib for 1 h before H_2_O_2_ treatment. Following incubation at 37 °C, cells were washed once with 4 °C PBS in a cold room. Untreated cells were washed twice at 4 °C with cold PBS. Cells were pelleted at 4 °C, and ADP-ribosylated peptides were enriched as described previously (41, 42, 43). In brief, cell pellets were lysed by adding 10 pellet volumes of guanidinium-HCl lysis buffer, vigorously shaking and vortexing in alternate steps, and snap freezing in liquid nitrogen. Lysates were slowly thawed at room temperature, treated with 5 mM TCEP (C4706, Sigma Aldrich) and 5 mM CAA (22790, Sigma Aldrich), and sonicated on ice for 45 s at 60% amplitude. Lysates were incubated with Lys-C endopeptidase (1:200 w/w; Wako Chemicals) for 3 h, diluted to 1.5 M guanidine-HCl with 50 mM ABC, and incubated overnight with modified sequencing-grade Trypsin (1:200 w/w; Sigma Aldrich). TFA was added to lysates at 0.5% v/v to inactivate proteases and lysates were centrifuged at high speed to remove precipitates. Peptides were purified using reverse-phase Sep-Pak C18 cartridges (WAT051910; Waters) according to manufacturer’s instructions and eluted in 30% ACN in 0.1% TFA. At this point, 5% of each sample was set aside for phosphoproteome enrichment and MS analysis as described in the next section. The remaining 95% of each sample was frozen at −80 °C at least overnight and lyophilized for 96 h. Peptides were reconstituted in AP buffer (50 mM Tris-HCl (pH 8.0), 50 mM NaCl, 1 mM MgCl2, and 250 μM DTT), and equal peptide amounts for each parent cell line and corresponding OR cell lines weer aliquoted (2 mg for MDAMB436 cells and 1.5 mg for HCC1428 cells). Recombinant hPARG (1:10,000 w/w, from Prof. Michael Höttiger) was added to each sample and incubated overnight at room temperature with gentle shaking to digest ADP-ribose polymers into monomers, and precipitates were removed by centrifuging at 4 °C for 30 min at 4250×*g*. GST-tagged Af1521 macrodomain beads, produced in-house using BL21(DE3) bacteria and coupled to glutathione Sepharose 4B beads (Sigma-Aldrich), essentially as previously described, were added to peptides (100 μl dry beads per 10 mg sample) and incubated in a head-over-tail mixer at 4 °C for 3 h to enrich for ADPr peptides. Beads were washed two times with ice-cold AP buffer, twice with ice-cold PBS with 250 μM DTT, and twice with ice-cold water. ADPr peptides were eluted with 0.15% TFA, centrifuged through 0.45 μM spin filters, and centrifuged again through 100 kDa cut-off spin filters (Vivacon). Peptides were desalted and fractionated on stage tips at high pH into two fractions. After loading onto stage tips for high pH fractionations, the flowthrough was collected and subjected to low pH stage tip purification. Each sample was prepared in quadruplicate.

#### LC-MS/MS of Proteome and ADP-Ribosylome Samples

Proteome MS data were collected with an Orbitrap Exploris 480 instrument (Thermo) using high-energy collisional dissociation (HCD) fragmentation in data-dependent acquisition (DDA) mode. ADPr MS data was collected with an Orbitrap Fusion Lumos Tribrid MS (Thermo) using electron-transfer/higher-energy collision dissociation (EThcD) fragmentation in DDA mode. Samples were analyzed on 15 to 20-cm long analytical columns with an internal diameter of 75 μm, packed in-house using ReproSil-Pur 120 C18-AQ 1.9 μm beads (Dr Maisch). Columns were connected to a nanoscale EASY-nLC 1200 liquid chromatograph (Thermo) and heated to 40 °C using a column oven, and peptides were eluted with a gradient of buffer A (0.1% formic acid) and buffer B (80% ACN in 0.1% formic acid). For proteome samples buffer B increased from 5% to 35% over the course of 50 min for the primary gradient, followed by an increase to 90% over 4 min, constant 90% for 2 min, decrease to 5% over 2 min, and 5% buffer B for 2 min (wash out). For high pH fractions of ADPr samples, buffer B increased from 3% to 38% over the course of 38 min in the primary gradient, followed by a 12-min wash-out. For low pH fractions of ADPr samples, buffer B was increased from 5% to 15% over the course of 22 min in the primary gradient, followed by an 18-min wash-out. Electrospray ionization (ESI) was achieved using a NanoSpray Flex NG ion source (Thermo). Spray voltage was set to 2 kV, capillary temperature to 275 °C, and RF level to 40%. Full scans were performed at 120,000 resolution, with a scan range of 300 to 1750 m/z and maximum injection time set to auto. The normalized AGC target was 200 for the proteome and 150 for ADPr samples. For proteome samples, the precursor isolation width was set to 1.3 m/z, normalized AGC target set to “200”, and precursors were fragmented using HCD with a normalized collision energy (NCE) of 25%. Top 18 precursors with charge states from 2 to 6 were isolated for MS/MS analysis, with 60 s dynamic exclusion. MS/MS spectra were measured in the Orbitrap at 15,000 scan resolution, and maximum precursor injection time was set to auto. Precursor isolation width for ADPr samples was 1.3 m/z, normalized AGC target was “400”, and precursors were fragmented using EThcD with 20% NCE. The top three precursors with charge states 3, 4, or 5 were isolated for MS/MS analysis, with 60 s dynamic exclusion. MS/MS spectra were measured in the Orbitrap t 60,000 scan resolution, with a maximum precursor injection time of 1000 ms.

### Phosphoproteome Mass Spectrometry

#### Phosphopeptide Enrichment

For phosphoproteome analysis, 50 or 35 μg of digested peptides prepared as described above were used for phosphopeptide enrichment of MDAMB436 and HCC1428 samples, respectively. Prior to enrichment, peptides were concentrated in a SpeedVac centrifuge to a volume of 20 μl and resuspended in 200 μl Loading buffer (80% ACN, 5% TFA, and 0.1 M glycolic acid). Enrichment of phosphopeptides was performed in a 96-well plate format on a KingFisher Flex robot (Thermo Fisher Scientific) essentially as described in Bekker-Jensen *et al*. (44), using 5 μl of ZrIMAC-HP beads (MagResyn, Resyn BioSciences) per sample. For all samples, two sequential rounds of enrichments were performed using the same buffers. Briefly, the 96-well comb was stored in plate #1, ZrIMAC-HP beads in 100% ACN in plate #2 and Loading buffer in plate #3. Plate #4 was the sample plate, while plates #5 to 7 contained the washing buffers in the following order: washing buffer 1 (Loading buffer), washing buffer 2 (80% ACN, 1% TFA), and washing buffer 3 (10% ACN, 0.2% TFA). Phosphopeptides were eluted in 200 μl 1% Ammonia (plate #8) and acidified with 10% TFA to pH < 3. Samples were filtered using a 0.45 μm MultiScreenHTS HV Filter Plate and centrifuged for 1 min at 500 × G, prior to loading the eluate containing enriched phosphopeptides onto Evotips, according to the instructions of the manufacturer.

#### LC-MS/MS of Phosphopeptides

Samples from phosphopeptide enrichment were analyzed on the EvoSep One system (using the pre-programmed 40 samples per day gradient) online coupled to an Orbitrap Exploris 480 MS through a Nanospray Flex ion source (Thermo Fisher Scientific). Peptides were separated on an IonOpticks Aurora column (15 cm, 150 μM inner diameter with 1.6 μm reversed-phase C18 beads) with column temperature maintained at 50 °C by an integrated column oven (PRSO-V2, Sonation GmbH). Phosphoproteome analysis was performed using data-independent acquisition (DIA) with HCD fragmentation, in positive polarity mode with spray voltage at 1.8 kV, RF level at 40, and heated capillary temperature at 275 °C. For DIA analysis, full scan resolution was set to 120,000 at m/z 200, and full scan AGC target was 300% with an injection time of 45 ms. The mass range was set to m/z 350 to 1400. AGC target value for fragment spectra was 1000%. For DIA phosphoproteome analysis, 26 windows of 27 m/z with an overlap of 1 m/z scanning from 472 to 1143 m/z were used. Resolution was set to 30,000 with an injection time of 54 ms, and normalized collision energy was set to 27%.

### Bioinformatic Analysis of Proteome Data

The raw MS data was analyzed using MaxQuant software version 2.0.3.1 with default settings unless indicated. A human fasta file downloaded on 11.03.2021 from Uniprot.org, containing 75,778 entries (SwissProt and TrEMBL, reviewed and unreviewed entries), as well as a fasta file with 245 common mass spectrometry contaminants provided by default in MaxQuant, were used to generate a theoretical spectral library. Cysteine carbamidomethylation was set as a fixed modification, Methionine oxidation and N-terminal acetylation were set as variable modifications, and the maximum modifications per peptide was 5. Up to two missed cleavages were allowed. The mass tolerance was set to ±20 for the first search and ±4.5 ppm for the main search. The false discovery rate (FDR) was set to 0.01 at the peptide and protein levels. To quantify peptide and protein amounts, label-free quantification (LFQ) via Fast LFQ (45) was enabled with normalization type set to classic. The LFQ minimum ratio count was set to two in group-specific parameters. Match between runs was enabled with default parameters. The list of identified proteins in the proteinGroups.txt file was further filtered to remove potential contaminants, reverse hits, proteins only identified by site, and proteins quantified with LFQ intensity in fewer than three replicates of at least 1 cell line.

### Bioinformatic Analysis of ADP-Ribosylome Data

The raw MS data was analyzed using MaxQuant version 1.5.3.30 with default settings unless indicated. A theoretical spectral library was generated using a human fasta file downloaded on 11.03.2021 from Uniprot.org, containing 75,778 entries (SwissProt and TrEMBL, reviewed and unreviewed entries), as well as the Maxquant default contaminant fasta file (245 entries). Methionine oxidation, N-terminal acetylation, cysteine carbamidomethylation, and ADPr on cysteine, aspartic acid, glutamic acid, histidine, lysine, arginine, serine, threonine, and tyrosine residues were set as variable modifications, and the maximum number of modifications per peptide was set to 5. The maximum allowed missed cleavages was set to 4. The mass tolerance was set to ±20 and ± 4.5 ppm for the first search and the main search, respectively. The FDR was set to 0.01 at peptide, protein, and site levels. Label-free quantification by fast LFQ was enabled and normalization type set to none. Match between runs was enabled with a default parameters. To ensure proper identification and localization of ADPr sites, the MaxQuant-generated ADPr sites table was further manually filtered with the statistical software Perseus (46) and Microsoft Excel to remove contaminants and reverse hits; to exclude peptide-spectrum matches (PSMs) with more than one ADPr modification; and to only include ADPr site assignments with localization probability above 0.9 for site identification and above 0.75 for quantification. Intensity values were then manually mapped from the evidence.txt to the ADPr sites table based on localized PSMs only, to exclude non-localized or poorly localized evidences. ADPr sites quantified with LFQ intensity in fewer than three replicates of at least one condition were removed.

### Bioinformatic Analysis of Phosphoproteome Data

All DIA raw files were analyzed using Spectronaut with a library-free approach (directDIA). A theoretical library was generated using a human fasta file downloaded on 18.11.2020 from Uniprot.org, containing 21,074 entries (SwissProt and TrEMBL, reviewed entries) as well as a fasta file with 246 common mass spectrometry contaminants. Cysteine carbamidomethylation was set as a fixed modification, Methionine oxidation, N-terminal acetylation, and phosphorylation on Serine, Threonine, and Tyrosine were set as variable modifications, and up to five modifications per peptide were allowed. The mass tolerance was set to ±40 ppm for both precursor and fragment ions. FDR was set to 0.01 at the PSM, peptide, and protein levels. Label-free quantification (LFQ) was performed using MaxLFQ (45) (spectronaut default). PTM localization filter was enabled, and PTM localization score cutoff was set to 0.75. Phosphopeptide quantification data were collapsed to site information with Perseus (V1.6.5.0) using the Perseus plugin as previously described (44). After processing, the list of identified phosphorylation sites was filtered to remove potential contaminants as well as sites where peptide LFQ intensities, a measure of peptide amounts, were quantified in fewer than three replicates of at least 1 cell line. To remove artefactual intensities assigned during Spectronaut processing, the phospho sites table was “censored” by replacing log-transformed intensity values less than 0 with NA. The phospho sites were row-wise median-centered per sample as follows: the mean of sample medians was calculated, and each sample median was subtracted from this value to generate a normalization factor for each sample. Individual intensities were then subtracted from the corresponding normalization factors.

### NAD Metabolite Measurements

#### Sample Preparation

Olaparib-sensitive BRCA1 mutant MDAMB436 and BRCA2 mutant HCC1428 cells, along with three OR cell lines derived from each parent cell line, were used. Experimental blanks consisted of wells with media but no cells. Media (∼500 μl) was collected from cells and stored at −80 °C. Adherent cells were rinsed with 1 ml/well PBS, and 360 μl of 90% methanol containing 5 μg/ml internal standard mix (NA-d4, NAD-d4, NADH-d4, and Nam-d4) was added to quench cells. Cells were scraped, transferred to microcentrifuge tubes, and combined with 90 μl 100 mM formic acid. Tubes were repeatedly snap-frozen in liquid nitrogen and vortexed. After the third cycle, 50 μl of 15% ammonium carbonate was added for neutralization. Samples were incubated on ice for 1 h, centrifuged at 13,000 rpm for 15 min at 4 °C, and stored at −80 °C.

#### LC-MS/MS Analysis

Samples were thawed, vortexed, and 50 μl was aliquoted into LC vials with inserts. Quality control pools were created by combining 20 μl from each sample type. Extraction and solvent blanks were included. In a second analysis, 200 μl of each extract was dried under nitrogen at 30 °C and resuspended in 50 μl of 90% methanol for instrument analysis.

Calibration standards were prepared using a mix of adenosine diphosphate ribose (ADPR), NAD, NADH+, and Nam in methanol, with concentrations determined based on the measured levels in pooled samples. Internal standards (10 μg/ml NA-d4, NAD-d4, NADH-d4, Nam-d4) were added to all calibration points and extracted similarly to the cell samples. A second calibration curve for nicotinic acid (NA), nicotinamide riboside (NR), nicotinic acid adenine dinucleotide (NAAD), nicotinamide mononucleotide (NMN), and nicotinic acid mononucleotide (NAMN) was prepared and up-concentrated post-extraction by drying and resuspension in a reduced volume. LC-MS/MS analysis was performed using a Waters Premier Acquity UPLC coupled to a Waters Xevo TQ-XS triple quadrupole mass spectrometer. Randomized samples were separated on a Waters Acquity Premier BEH Amide HILIC column (2.1 × 150 mm, 1.7 μm), maintained at 40 °C. Mobile phase A consisted of 10 mM ammonium acetate in H2O with 5 μM medronic acid, and phase B was 10 mM ammonium acetate in 90% ACN with 5 μM medronic acid. A gradient method with a total run time of 7.5 min was used, starting at 90% B, transitioning to 55% B, and returning to initial conditions. The mass spectrometer operated in positive ion mode with electrospray ionization. Capillary voltage was 0.6 V, cone voltage was 15 V, desolvation temperature was 600 °C, and gas flow was 1000 L/h. Multiple reaction monitoring (MRM) transitions for each analyte were optimized, and analyte-specific collision energies were applied.

#### Data Processing

Data were analyzed using Skyline (v. 24.1.0.199). Molecule transition lists with retention times, parent ions, and fragment ions were uploaded, and peaks were automatically picked and integrated. Calibration curves were plotted using peak area ratios of analytes to internal standards, weighted (1/x^2^). Calibration points with >15% concentration error were excluded. Quality control was assessed using pooled samples, with relative standard deviations under 25%. Limits of quantitation (LoQ) were established based on a signal-to-noise ratio of 10.

Concentrations of NR, NAD, ADPR, Nam, and NADH were calculated using calibration curves, while NMN, NAMN, NA, and NAAD were below LoQ. Raw values are presented Supplemental Table 5.

### Quantification and Statistical Analyses

Statistical analysis of MS data, including principal component analysis (PCA) and volcano plot analysis was performed using Perseus software (46). Proteome volcano plot significance is based on two-sided T-tests with 250 randomizations and an S0 of 0.1 (47), and *p*-values were adjusted for multiple testing by setting a 0.05 false discovery rate (FDR) cutoff. RNA sequencing volcano plot significance, calculated in DESeq2 using default methods, are based on the Wald test and *p*-values were adjusted for multiple testing with 0.05 FDR control using the Benjamini-Hochberg method. Ranked Gene Set Enrichment Analysis (GSEA) on differentially expressed proteome data was performed using fGSEA implemented in R (48). Functional annotation of volcano plot significant proteins was performed using Enrichr implemented in the GSEApy python package (49). Cytotoxicity curves for Olaparib were calculated and average CC_50_ values were interpolated in Graphpad Prism using the [Inhibitor] vs. normalized response—Variable slope nonlinear fit formula. Bar plots, pie charts, and interleaved scatter plots showing individual protein, phosphorylation, or ADPr intensities, were generated with GraphPad Prism. *p*-values shown for individual protein, phosphopeptide, and metabolite intensities were calculated with GraphPad Prism using one-way ANOVA with multiple comparisons, where intensities for each OR cell line were tested against the corresponding parent cells. *p*-values were adjusted using the Dunnett test for multiple testing correction. UpSet plots were generated with the ComplexHeatmap R package. Box plots, and heatmaps were generated with custom R scripts. STRING functional association networks were generated using the Cytoscape app (version 3.10.1) (50) using Uniprot IDs, and the StringDB score cutoff, which is the confidence score assigned to each protein-protein interaction, indicating the likelihood of the interaction being biologically relevant, was set to 0.7.

## Results

### Olaparib-Resistant Cell Lines Derived From BRCA1/2 Mutant Breast Cancer Cells Express ADPr Signaling Enzymes and Stimulate ADPr in Response to H_2_O_2_ Treatment

**Fig. 1:**
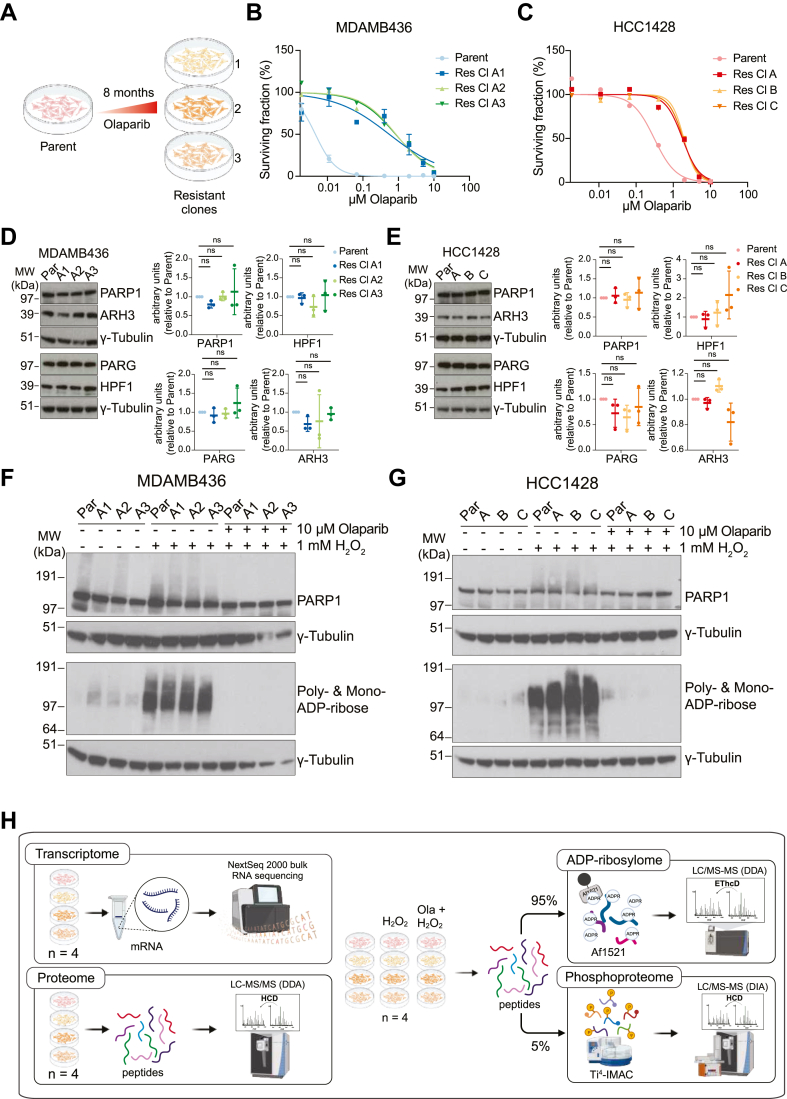
**Olaparib-resistant clones of BRCA1/2 mutant breast cancer cells express ADPr signaling enzymes and stimulate ADPr in response to H_2_O_2_ treatment.***A*, generation of Olaparib resistance in PARPi-sensitive breast cancer cell lines Three Olaparib-resistant (OR) cell lines each were generated by treating Olaparib-sensitive MDAMB436 and HCC1428 cells continuously with increasing concentrations of the drug over an 8-month period. *B* and *C*, colony formation assay curves show percentage survival relative to DMSO-treated controls for parent cells and three resistant clones in MDAMB436 cells (*B*) and HCC1428 cells (*C*). Cells were treated with a range of Olaparib concentrations for 15 days (MDAMB436 cells) or 19 days (HCC1428 cells). Error bars represent the standard deviation of three technical replicates per drug concentration. CC_50_ values for three biological replicates are shown in Table 1. Cytotoxicity curves and average CC_50_ values were calculated in Graphpad Prism, using the [Inhibitor] vs. normalized response – Variable slope nonlinear fit formula. *D* and *E*, Representative western blots (WBs) showing expression of ADPr writer enzyme PARP1 and its cofactor HPF1, and eraser enzymes PARG and ARH3 in parent and OR clones of MDAMB436 cells (*D*) and HCC1428 cells (*E*). Quantification of WB protein abundance of PARP1, HPF1, PARG, and ARH3 in parent and OR cells, normalized to ɣ-tubulin loading control, are to the right of each WB. n = 3 technical replicates. Error bars represent the standard deviation from the mean (SD). *F* and *G*, WB show PARP1 and ADPr levels in MDAMB436 (*F*) and HCC1428 (*G*) parent and resistant clones in basal conditions, after 10-min treatment with 1 mM H_2_O_2_, and after 1 h pretreatment with 10 μM Olaparib before H_2_O_2_ treatment. *H*, schematic illustration of workflow used for transcriptome, proteome, ADP-ribosylome, and phosphoproteome characterization of parent cells and OR clones. For proteome and ADP-ribosylome samples, the mass spectrometer was operated in Data-Dependent Acquisition (DDA) mode for proteome and ADP-ribosylome samples, and in Data-Independent Acquisition (DIA) mode for phosphoproteome samples. Higher energy collision-induced dissociation (HCD) fragmentation was used for proteome and phosphoproteome samples, and Electron-Transfer/Higher-Energy Collision Dissociation (EThcD) fragmentation was used for ADPr samples. 4 biological replicates were analyzed for all cell lines and treatments.

To characterize the changes leading to Olaparib resistance in breast cancer cells, we generated Olaparib resistance in Olaparib-sensitive BRCA1 mutant MDAMB436 and BRCA2 mutant HCC1428 cells by culturing them with increasing concentrations of PARPi (51, 52) (Fig. 1*A*). The starting Olaparib concentrations used were 5 nM for MDAMB436 cells and 0.2 μM for HCC1428 cells and correspond to the amount needed to inhibit the 50% of colony growth (CC_50_ values) in these cells as determined from Anagho *et al*., 2024 (30). Eight months later, the cells could tolerate 0.2 μM and 2 μM Olaparib, respectively, indicating acquired OR. We generated three OR cell lines from each parent cell line so that we could study whether resistance would develop through similar or diverging mechanisms. Increased Olaparib tolerance in each OR cell line relative to the parent cells was validated by colony formation assay (Fig. 1, *B* and *C*) and we found that the CC_50_ values of HCC1428 OR cell lines were approximately 3 to 6 times higher than parent cells. Surprisingly, the CC_50_ values of MDAMB436 OR cell lines were around 119 to 350 times higher (Table 1), which is similar to Olaparib CC_50_ values in BRCA wild-type breast cancer cells (30).

**Table 1:** Olaparib CC_50_ values of parent breast cancer cells MDAMB436 and HCC1428, and three OR cell lines generated from each cell line

Cell line	CC_50_ (μM) rep 1	CC_50_ (μM) rep 2	CC_50_ (μM) rep 3
MDAMB436 Parent	0.0046	0.0073	0.0099
MDAMB436 Res Cell Line A1	0.5527	2.5034	1.4963
MDAMB436 Res Cell Line A2	0.8560	1.1086	3.5025
MDAMB436 Res Cell Line A3	0.8361	2.4809	2.366
HCC1428 Parent	0.308	0.480	0.520
HCC1428 Res Cell Line A	1.768	2.245	1.421
HCC1428 Res Cell Line B	1.932	2.270	2.168
HCC1428 Res Cell Line C	1.853	2.439	2.007

CC_50_ values were calculated from colony formation assays using the log inhibitor response function in Graphpad Prism. Three technical replicates and three biological replicates were analyzed.

Because Olaparib targets PARPs and affects ADPr signaling, we determined expression levels of the involved enzymes, PARP1, its cofactor HPF1, and the glycohydrolases PARG and ADP-ribosylhydrolase 3 (ARH3), in the parent cells and OR cell lines by Western blot (WB). For both cell lines, levels of PARP1, PARG, HPF1, and ARH3 in OR cell lines were similar compared to parent cells (Fig. 1, *D* and *E*). Next, we assessed by WB whether ADPr is stimulated by H_2_O_2_ treatment and inhibited by Olaparib in OR cell lines. For this, cells were treated with 1 mM H_2_O_2_ for 10 min to stimulate ADPr, or pre-treated with 10 μM Olaparib for 1 h to inhibit PARP activity, followed by H_2_O_2_ treatment. We found that ADPr was stimulated by H_2_O_2_ treatment and blocked by Olaparib pre-treatment in all OR cell lines (Fig. 1, *F* and *G*), suggesting that DNA damage-stimulated ADP-ribosylation is still functional. Interestingly, slightly higher ADPr signal could be detected in the HCC1428 OR cell lines compared to the parent cell line, whereas PARP automodification was decreased in the MDAMB436 OR cell lines (Fig. 1*G*).

To better understand the changes associated with Olaparib resistance development in MDAMB436 and HCC1428 cells, we undertook a systems-level characterization of these cell lines. This involved sequencing poly-A-enriched RNA in parent and OR cell lines, as well as assessing protein expression through tandem mass spectrometry coupled to liquid chromatography (LC-MS/MS) (Fig. 1*H*). To gain insight into changes in regulation of post-translational modifications (PTMs), we also characterized ADP-ribosylation and phosphorylation in cells treated with 1 mM H_2_O_2_ for 10 min, as well as cells pretreated with 10 μM Olaparib for 1 h before H_2_O_2_ treatment (Ola + H_2_O_2_), with untreated cells as controls.

### BRCA1 Expression is Regained in all Olaparib-Resistant MDAMB436 Cell Lines

**Fig. 2:**
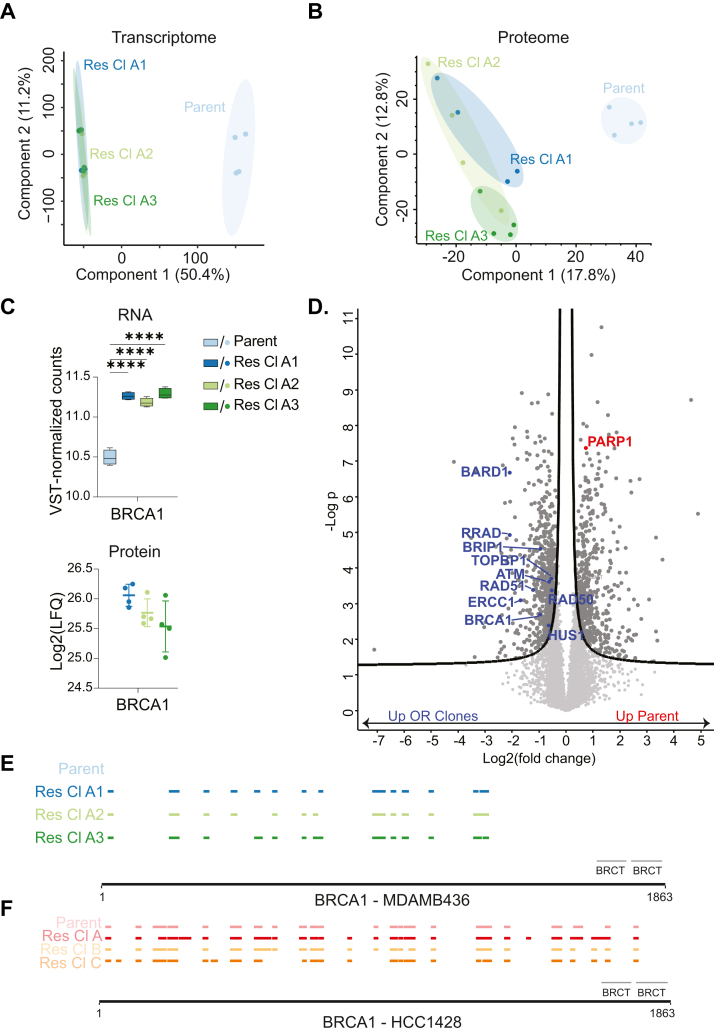
**BRCA1 expression is regained in OR MDAMB436 cells.***A*, principal component analysis (PCA) plot from DeSeq2 of the gene counts based on RNA sequencing of MDAMB436 Parent and OR cells. *B*, PCA plot based on MS-derived protein label-free quantification (LFQ) intensities. *C*, BRCA1 mRNA levels (*top*) and protein levels (*bottom*). *D*, Volcano plot shows the log2 fold change and -log *p* values between the mean LFQ intensity of the parent cells and the mean of the three OR cell lines, with proteins significantly upregulated in *dark gray*. Proteins significantly upregulated in OR cells are on the *left* and proteins significantly upregulated in parent cells are on the *right*. Proteins of the Reactome pathway “HDR Through Single Strand Annealing” that were significantly upregulated in OR cell lines are labeled in *blue*, and PARP1 is labeled in *red*. Significance is based on two-sided T-tests between parent *versus* all three OR cell lines, and *p*-values were adjusted by setting an FDR of 0.05. E-F. Positions of MS proteome-detected BRCA1 peptides in MDAMB436 (*E*) and HCC1428 (*F*) Parent and OR cells, with BRCT domain positions indicated in *gray*.

To better understand the changes leading to OR in MDAMB436 cells, we explored transcript and protein expression in the parent and OR cells. From a quantitative proteomics analysis, we identified an average of 80,000 unique peptides per cell line (Supplemental Fig. S1*A*), corresponding to at least 6600 unique proteins (Supplemental Fig. S1*B*). Principal component analysis (PCA) of mRNA-sequencing and proteome data revealed that the OR cell lines formed a distinct cluster, which was separate from the parent cells (Fig. 2, *A* and *B*), suggesting that they are more similar to each other. This was also shown with Pearson Correlations of the proteome data (Supplemental Fig. S1*C*).

Strikingly, Olaparib CC_50_ values of OR MDAMB436 cells were found to be similar to BRCA wild-type cells. In cells with BRCA loss-of-function mutations, resistance to Olaparib can be achieved by the acquisition of secondary mutations that restore protein functionality (32). In fact, 50% of breast cancer patients with BRCA mutations acquire resistance through such reversion mutations (53). We therefore hypothesized that BRCA1 expression was restored in OR MDAMB436 cells. Transcriptome analyses showed that BRCA1 RNA levels were indeed significantly upregulated in all three OR cell lines compared to parent cells (Fig. 2*C*, top panel). Moreover, BRCA1 protein was detected in all three OR cell lines but not in the parent cells (Fig. 2*C*, bottom panel). T-tests could not be calculated on the protein level between the parent cells and OR cell lines due to the missing values, but volcano plots generated using imputed missing values showed that BRCA1 was significantly upregulated in OR cells compared to parent cells (Fig. 2D, Supplemental Fig. S1*D*).

Next, we investigated sequence and peptide changes in the BRCA1 protein that could lead to the reestablishment of expression in OR cells. In MDAMB436 cells, the BRCA1 gene carries a point mutation at position 5396 + 1G > A, specifically in the splice donor site of exon 20 (54). This mutation leads to transcription of additional codons in the intron, eventually reaching a premature stop codon, resulting in expression of a protein variant truncated at the BRCT domain. Consequently, these variants have protein-folding defects and are unstable (55). In all replicates of both parent and resistant clones, we identified the mutation known to affect splicing in this cell line in exon 18, c.1965+1G > A for RefSeq transcript NM_007298 (corresponding to exon 20, c.5340+1G > A in NM_007300). This variant most likely corresponds to the mutation described above; however, its annotation corresponds to older transcript annotations. The variant appeared to be homozygous, with allelic depths ranging from 3 to 11 reads across the 16 samples. (Supplemental Fig. S1*E*). This suggests that the OR cells still contain the original BRCA1 mutation. From our proteome analysis, no peptides corresponding to the BRCT domains in BRCA1 were identified in any of the OR cells (Fig. 2*E*), supporting the RNA sequencing data. In contrast, peptides corresponding to both BRCT domains were identified in all HCC1428 cell lines analyzed (Fig. 2*F*). Moreover, from a variant calling analysis of the transcriptomes, we did not find additional mutations in BRCA1 or other known DNA damage repair proteins that could explain its increased levels in the OR cells (Supplemental Fig. S1*F*). Taken together, the data strongly suggest that all three OR MDAMB436 cell lines have regained expression of BRCA1, which would explain their resistance to Olaparib. However, this doesn’t appear to be caused by RNA or peptide changes in the BRCA1 protein itself. It is possible that increased interactions between BRCA1 and other proteins may account for its increased stability, but this remains to be investigated. BARD1 and Other HDR-Related Proteins are Upregulated in OR MDAMB436 Cell Lines, Whereas PARP1 Expression is Decreased

**Fig. 3:**
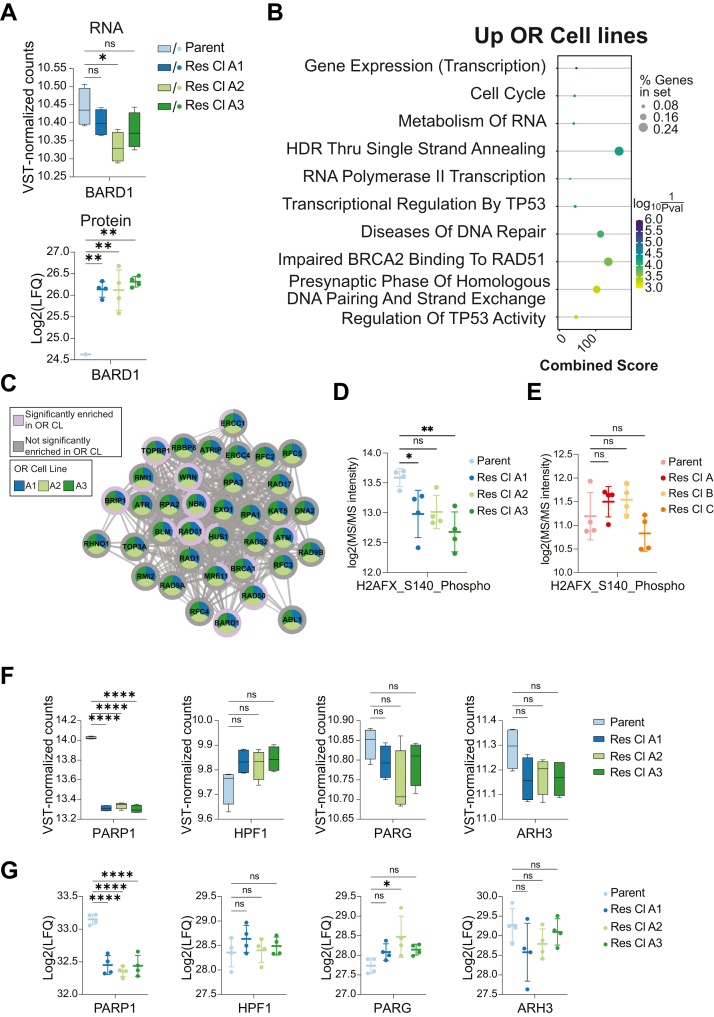
**Homology-directed repair protein levels, including BARD1, are increased, ɣH2AX is decreased, and PARP1 expression is downregulated in OR MDAMB436 cells with regained BRCA1 expression.***A*, BARD1 mRNA levels (*top*) and protein levels (*bottom*) in MDAMB436 parent and OR cells. *B*, functional enrichment performed using Enrichr in GSEApy with proteins significantly upregulated in OR cell lines as foreground and the human genome as background, and the dot plot shows the top 10 enriched Reactome pathways. The size of the dots represents the percentage of genes in the input set, and dots are colored by log10 (1/*p*-value). The combined score is the log *p*-value calculated from a Fisher exact test multiplied by the Z-score of the deviation from the expected rank. *C*, functional association network generated with STRING shows proteins belonging to the Reactome term “HDR Through Single Strand Annealing”. Proteins significantly enriched in each OR cell line, based on two-sided T-tests, are shown in purple (see also Supplemental Fig. S1*E*). *D* and *E*, Log2-transformed and median-normalized intensities of S140-phosphorylated histone γH2AX in MDAMB436 (*D*) or HCC1428 (*E*) parent cells and OR cell lines in untreated conditions, detected by MS of enriched phosphopeptides. *F*, Boxplots showing mRNA levels of PARP1, HPF1, PARG, and ARH3 in MDAMB436 parent and OR cells. *G*, MS-detected protein levels of PARP1, HPF1, PARG and ARH3 in MDAMB436 parent and OR cells. In (*D*), (*E*), (*F*), and (*G*), Statistical significance is based on one-way ANOVA with multiple comparisons, of mean intensity values for parent *versus* mean intensity values for each OR cell line. *p*-values were adjusted using the Dunnett test for multiple testing correction. ns = not significant. ∗ = *p* < 0.05; ∗∗ = *p* < 0.01; ∗∗∗ = *p* < 0.001; ∗∗∗∗ = *p* < 0.0001. Error bars represent the SD. n = 4 technical replicates per condition.

Next, we investigated the effects of restored BRCA1 expression in OR MDAMB436 cells by analyzing RNA and protein changes in genes functionally associated with BRCA1 in OR MDAMB436 cells. The BRCA1-associated RING domain protein 1 (BARD1), which heterodimerizes with and is essential for BRCA1 stability, and whose stability in turn also depends on the presence of BRCA1 (56), was significantly increased in the OR cell lines at the protein level (Fig. 3*A*, bottom). In contrast, BARD1 mRNA levels were not significantly different in OR A1 and A3, and even decreased in OR A2 compared to the parent cell line (Fig. 3*B*, top), suggesting that BARD stability is regulated on the protein level. Moreover, like BRCA1, we did not find any mRNA sequence changes in BARD1 that could explain the protein-level increases in the OR cells. This suggests that the increase in BARD1 protein levels in OR cells might be due to its stabilization in the presence of BRCA1 protein, and this is in agreement with published reports that BARD1 is an obligate heterodimeric partner of BRCA1 (57, 58, 59, 60).

Next, we performed functional annotation of the proteins significantly upregulated in all three OR cell lines relative to the parent cells from the volcano plot analysis (Fig. 2*D*) and found that “HDR Through Single Strand Annealing” was the fourth significantly enriched Reactome term (Fig. 3*B*). Of the 37 genes in this pathway, 14 are significantly enriched in one or more of the OR cells (Fig. 3*C*). An example includes RAD51, which is recruited to DNA double strand breaks by the BRCA1-BARD1 heterodimer as a crucial early step of HDR (60). The fact that not just BRCA1 but also the proteins that associate with it during HDR are significantly enriched in the proteome strongly suggests that HDR functionality is regained in MDAMB436 OR cell lines due to BRCA1 becoming stably expressed again.

Given the increased levels of BRCA1 and HDR proteins in OR MDAMB436 cells, we hypothesized that OR cells carry a reduced burden of DNA damage. To investigate this, we assessed our phosphoproteomics data for histone H2AX phosphorylation at serine 140 (histone γH2AX; based on the Uniprot numbering scheme), a proxy for DNA double-strand break detection and repair (61). We found that histone γH2AX levels were lower in OR cells compared to parent cells (Fig. 3*D*), and this was statistically significant in OR cell lines A1 and A3. In contrast, γH2AX levels were similar across HCC1428 parent and OR cells (Fig. 3*E*).

Finally, we investigated whether these changes affected the expression of ADPr signaling enzymes. Transcriptome and proteome analysis showed that PARP1 RNA and protein levels were significantly downregulated in OR cell lines compared to parent cells (Fig. 3, *F* and *G*), while PARG protein levels were significantly higher in OR A1 cells compared to parent cells (Fig. 3*G*), and RNA and protein levels of the other ADPR signaling proteins HPF1, ARH3, and PARG were not significantly different compared to parent cells. Together with the observation that the overall ADPr levels in the OR cell lines after DNA-damage induction with H_2_O_2_ remained unchanged (Fig. 1*F*), our data suggest that the acquisition of OR does not generally affect the ADPr signaling pathway, but rather specifically targets PARP1 expression.

Together, the data suggest that all three MDAMB436 OR clones have acquired similar CC_50_ values to those seen in BRCA WT breast cancer cell lines (62) by reestablishing BRCA1 expression and HDR functionality and with that eliminating the synthetic lethal effect that its absence has with PARP inhibition. The cells have also downregulated PARP1 expression, potentially mitigating toxic effects of PARP trapping resulting from culturing them in the presence of Olaparib (63).

### The BRCA2 Mutation is Not Reverted in HCC1428 OR Cell Lines

**Fig. 4:**
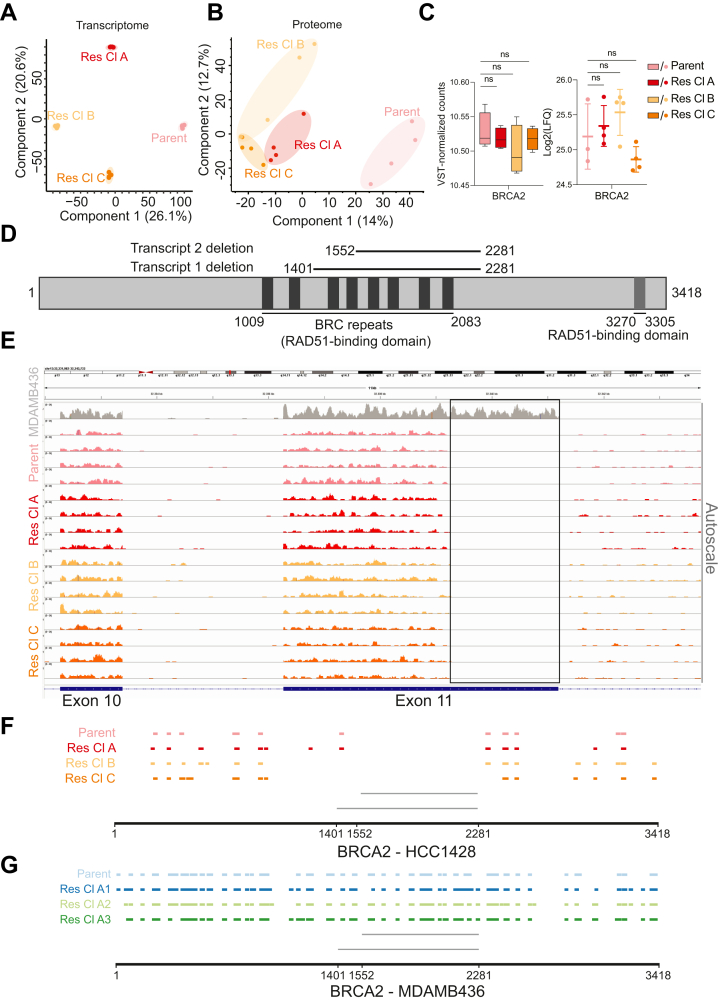
**The BRCA2 mutation is not reverted in Olaparib-resistant HCC1428 cells.***A*, PCA plot from DeSeq2 of RNA sequencing-based gene counts of HCC1428 Parent and OR cells. *B*, PCA plot based on MS-derived protein LFQ intensities. *C*, BRCA2 mRNA levels (*left*) and protein levels (*right*). Statistical significance is based on one-way ANOVA with multiple comparisons of mean LFQ intensity values for parent *versus* mean LFQ intensity values for each OR cell line. *p*-values were adjusted using the Dunnett test for multiple testing correction. ns = not significant. ∗ = *p* < 0.05; ∗∗ = *p* < 0.01; ∗∗∗ = *p* < 0.001; ∗∗∗∗ = *p* < 0.0001. Error bars represent the standard deviation from the mean. *D*, schematic of BRCA2 showing 8 BRC repeats from amino acid (aa, *black*) 1009 to 2083 (65, 66), and additional C-terminal region (*dark gray*) at aa 3270 to 3305 (97, 98), where RAD51 binds to BRCA2. Deletion regions for the two BRCA2 transcripts expressed in HCC1428 cells (Transcript 1, aa 1552–2281; Transcript 2, aa 1401–2281) are indicated (64). The schematic is not drawn to scale. *E*, IGV tracks of BRCA2 mRNA peaks of HCC1428 Parent and OR cells at Exon 10 and at Exon 11, where the BRCA2 deletion region is located. The IGV trace for the corresponding region in MDAMB436 parent cells, where full-length BRCA2 is expressed, is shown in *gray*. *F-G*, positions of MS proteome-detected BRCA2 peptides in HCC1428 (*F*) and MDAMB436 (*G*) parent and OR cell lines. *Gray* lines indicate deletion regions for transcripts 1 and 2.

We next analyzed transcriptome and proteome changes leading to OR in HCC1428 cells. On RNA- and protein-level PCAs, each cell line formed a distinct cluster (Fig. 4, *A* and *B*), indicating that the cells are more different to each other compared to the MDAMB436 OR cell lines. Nevertheless, protein-level PCAs showed that the OR HCC1428 cells were still more similar to each other than to the parent cells. The more distinct clustering of OR HCC1428 cells could be explained by the fact that while all MDAMB436 OR cell lines acquired PARPi resistance largely through reestablishment of BRCA1 expression, this is an unlikely scenario for the BRCA2 mutant HCC1428 cells, due to the nature of this mutation.

HCC1428 cells have a mutation in BRCA2 that leads to the expression of two alternatively spliced transcripts (64): Transcript 1 has a deletion in nucleotides 4430 to 7069 while Transcript 2 has a deletion in nucleotides 4883 to 7069 (Fig. 4*D*). The transcripts encode a BRCA2 protein missing 881 and 730 amino acids, respectively, both in a region containing ssDNA binding domains, nuclear localization signals, and some BRC repeats, where RAD51 proteins bind, rendering the protein non-functional in HDR (64, 65, 66). Unsurprisingly, similar levels of BRCA2 mRNA and protein were detected in parent and OR HCC1428 cells (Fig. 4*C*). Moreover, RNA sequencing analysis showed that the deletion in Exon 11 was present in the parent cells and in all of the OR cells (Fig. 4*E*). In contrast, the BRCA2 transcript detected in MDAMB436 corresponds to the entire Exon 11. We also confirmed that the BRCA2 proteins detected in HCC1428 cells were missing peptides corresponding to the expected deletion in the parent and the OR cell lines (Fig. 4*F*), whereas several peptides from this region were detected in parent and OR MDAMB436 cells (Fig. 4*G*). These data illustrate that unlike MDAMB436 cells, the HCC1428 OR cell lines have not undergone a BRCA2 reversion mutation as a means of acquiring OR. Together with the more distinct PCA clustering of the individual OR cell lines, this suggests that OR in the HCC1428 cells was acquired by other means.

### Determinants of Olaparib Resistance in HCC1428 cells

#### Protein Levels of PARP1 Co-factor HPF1 are Increased in HCC1428 OR Cells

**Fig. 5:**
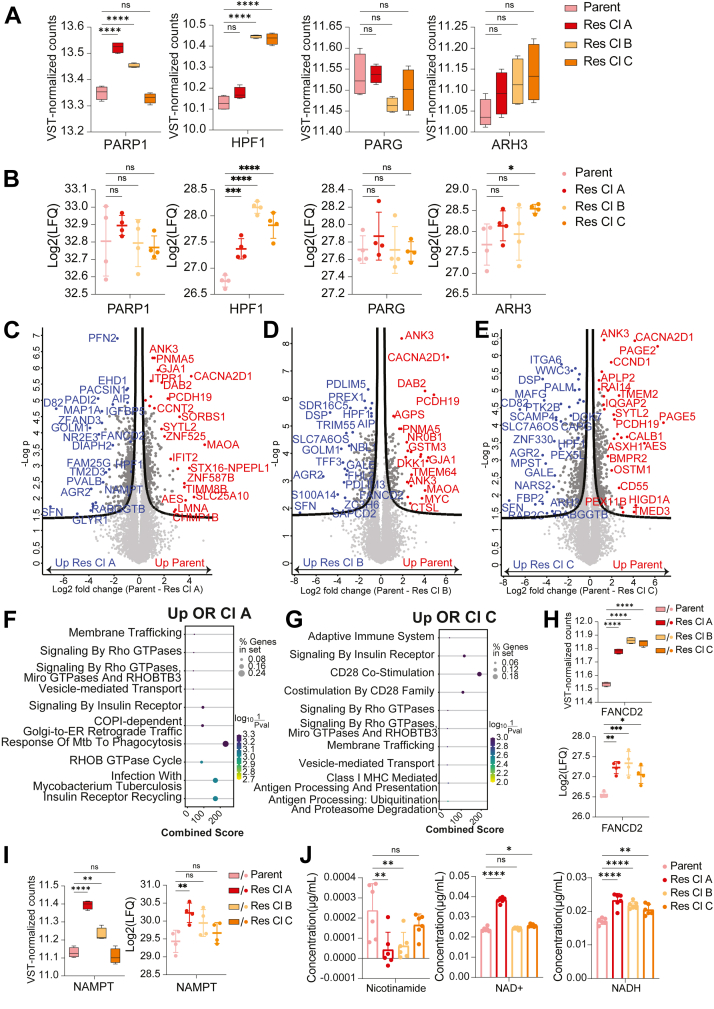
**Determinants of Olaparib resistance in HCC1428 cells.***A*, Boxplots showing mRNA levels of PARP1, HPF1, PARG, and ARH3 in HCC1428 parent and OR cells. *B*, MS-detected protein levels of PARP1, HPF1, PARG and ARH3 in HCC1428 parent and OR cells. *C-E*, Volcano plots show the log2 fold change and -log2 *p* values between the mean LFQ intensity of the parent cells and OR cell line A (*C*), B (*D*) or C (*E*), with the most differentially expressed colored and labeled in *blue* if up in OR cells or *red* if up in parent cells. *p*-values are based on two-sided T-tests between parent and each OR cell line, with 0.05 FDR. *F-G*, Functional enrichment on proteins significantly upregulated in Res Cl A relative to parent (*F*) from volcano plot C, or in Res Cl C relative to parent (*G*) from volcano plot E as foreground and the human genome as background. The dot plots show the top 10 significantly enriched Reactome pathways, with dot size representing the percentage of genes in the input set, and dots colored by log10 (1/*p*-value). The combined score is the Fisher exact test log *p*-value multiplied by the Z-score of the deviation from the expected rank. Functional annotation was performed using Enrichr in GSEApy. *H*, FANCD2 mRNA levels (*top*) and protein levels (*bottom*). *I*, NAMPT mRNA (*left*) and protein (*right*) expression HCC1428 parent and OR cells. *J*, Concentration of Nicotinamide, NAD+, and NADH in HCC1428 parent and OR cells, normalized to the average protein amount per cell line. N = 6 per cell line. In (*A*), (*B*), (*H*), (*I*), and (*J*), statistical significance is based on one-way ANOVA with multiple comparisons of mean variance-stabilizing transformation (VST)-normalized counts (RNA), LFQ intensity values (protein), or metabolite concentrations (*J*) for parent *versus* each OR cell line. *p*-values were adjusted using the Dunnett test for multiple testing correction. ns = not significant. ∗ = *p* < 0.05; ∗∗ = *p* < 0.01; ∗∗∗ = *p* < 0.001; ∗∗∗∗ = *p* < 0.0001. Error bars represent the standard deviation from the mean.

To find determinants of OR in HCC1428 cells, we started by analyzing expression levels of ADPr signaling enzymes: PARP1, its cofactor HPF1, and the glycohydrolases PARG and ARH3. In HCC1428 cells, WB analysis indicated similar expression levels of ADPr signaling enzymes across all cell lines (Fig. 1, *D* and *E*). Nonetheless, the more sensitive transcriptome and proteome analyses showed different results. On the RNA level, PARP1 expression was significantly higher in OR A and B cells, while HPF1 levels were significantly higher in OR B and C cells (Fig. 5*A*). PARG and ARH3 RNA levels, however, remained comparable. On the protein level, HPF1 was significantly higher in all three OR cell lines, while ARH3 was significantly higher in OR cell line C compared to parent cells (Fig. 5*B*).

#### Proteins Involved in Cancer Cell Migration, Metastasis, and Motility are Upregulated in OR Cell Lines

We next analyzed our MS proteome data to uncover pathway changes leading to OR in HCC1428 cells. From the MS-proteome analysis, we identified around 90,000 peptides per cell line on average, mapping to around 7100 proteins per cell line (Supplemental Fig. S2, *A* and *B*). High Pearson correlation coefficients (Supplemental Fig. S2*C*) also indicated high reproducibility between replicates.

To identify differentially expressed pathways in OR cells, we conducted ranked Gene Set Enrichment Analysis (GSEA) on the proteomes, comparing changes both across all OR cell lines relative to the parent cells and individually for each OR cell line relative to the parent cells (Supplemental Fig. S2*D*). We found that the same pathways were differentially regulated in each of the OR cells compared to the parent cells, as well as in all OR cells compared to parent cells. However, the comparisons of individual OR cell lines to the parent showed that the changes were only statistically significant in OR Cl A. Moreover, the enriched pathways have no obvious link to known DNA damage signaling or PARPi resistance pathways. We therefore took a different approach: we performed a volcano plot analysis to identify proteins significantly differentially expressed in each cell line compared to parent (Fig. 5, *C*–*E*) and then performed functional annotation on these significant proteins. Comparisons between the parent cells and Res Cl. A showed that various RNA processing and metabolism pathways were upregulated in parent cells (Supplemental Fig. S2*E*), while Rho GTPase signaling pathway proteins and insulin receptor signaling pathway proteins, including MAPK1 and MAPK3, were upregulated in Res Cl A (Fig. 5*F*). Similar pathways were also upregulated in Res Cl. C (Supplemental Fig. S2*F*, Fig. 5*G*), but no pathways were significantly enriched in Res Cl B. MAPK1 and MAPK3 signaling is known to affect cancer cell migration and metastasis (67), while Rho GTPases also affect cell motility (68), which may also not be directly linked to Olaparib resistance.

#### Expression of the Replication Fork Protection Protein FANCD2 is Increased in OR Cells

We then asked whether individual proteins known to be involved in PARPi resistance in BRCA mutant cells were differentially expressed in the OR HCC1428 cells. This includes proteins involved in restoring HDR functionality, reducing PARPi bioavailability, decreasing PARP trapping, or increasing replication fork protection (69). We found that levels of FANCD2, involved in protection of the replication fork (70) and known to have synthetic lethal interactions with BRCA2, were significantly higher in all OR cell lines compared to parent cells, both on the RNA and protein level (Fig. 5*H*, top and bottom panels, respectively).

#### Expression Changes in NAD + Salvage Pathway Enzyme NAMPT in Affects NAD + Metabolite Levels in HCC1428 OR Cell Lines

Because PARPs use NAD + as a substrate to make ADPr, and NAD+ is rapidly consumed when DNA damage is stimulated (71), and because HPF1 levels were higher in OR HCC1428 cell lines, we asked whether expression of proteins involved in NAD + metabolic pathways were altered. We found that the enzyme nicotinamide phosphoribosyltransferase (NAMPT) was significantly increased in OR cell lines A and B on the RNA level, and additionally in OR cell line A on the protein level (Fig. 5*I*). NAMPT plays a crucial role in the salvage pathway for NAD + biosynthesis by converting the byproduct of the PARP ADP-ribosylation reaction, nicotinamide, into nicotinamide mononucleotide (NMN), and is the rate-limiting enzyme in this pathway (33, 72). NMN is then converted to NAD+ (73) by nicotinamide nucleotide adenylyltransferases (NMNAT) 1, 2, or 3 (73), which localize to the nucleus, cytoplasm, and mitochondria, respectively (74, 75, 76).

To determine whether differences in NAMPT expression led to changes in metabolite levels, we measured the basal levels of metabolites involved in NAD biogenesis in HCC1428 cells. OR A cells, which had the highest increase in NAMPT levels compared to parent cells—and the only significant protein-level increase—showed the lowest nicotinamide levels and the highest NAD+ and NADH levels, all of which were statistically significant (Fig. 5*J*). In OR B cells, nicotinamide levels were significantly lower, and NADH levels were significantly higher. Similarly, OR C cells had significantly higher NAD+ and NADH levels, although nicotinamide levels were not significantly different. Overall, these metabolite-level changes corresponded to the observed protein-level changes in OR cells.

Interestingly, despite significant downregulation of NAMPT at both the RNA and protein levels in all OR MDAMB436 cells (Supplemental Fig. S2*G*), the metabolite levels showed weaker correlations. Nicotinamide levels were significantly reduced across all three OR cells, while NAD+ and NADH levels were significantly upregulated in OR A1 compared to parent cells (Supplemental Fig. S2*H*). Conversely, OR A2 and A3 exhibited decreased NAD+ and NADH levels, although the NAD+ change in OR A2 was not statistically significant. Nevertheless, these findings suggest that protein-level changes in NAD+ biogenesis enzymes have only modest effects on NAD metabolite levels.

In summary, these results show that since the large BRCA2 deletion was not reverted in the OR HCC1428 cells, a range of small-scale changes in individual proteins contributed to Olaparib resistance in HCC1428 cells, ranging from proteins involved in ADP-ribosylation to enhanced fork protection. Although OR cells also exhibit changes in pathways linked to motility, cell migration, and metastasis, this might be an effect of keeping the cells in culture for a long time rather than a specific OR mechanism.

### ADPr Response to H_2_O_2_-Induced DNA Damage is Homogeneous in MDAMB436 and HCC1428 Parent and OR Cells, With Modest Differences Corresponding to Protein-Level Changes

**Fig. 6:**
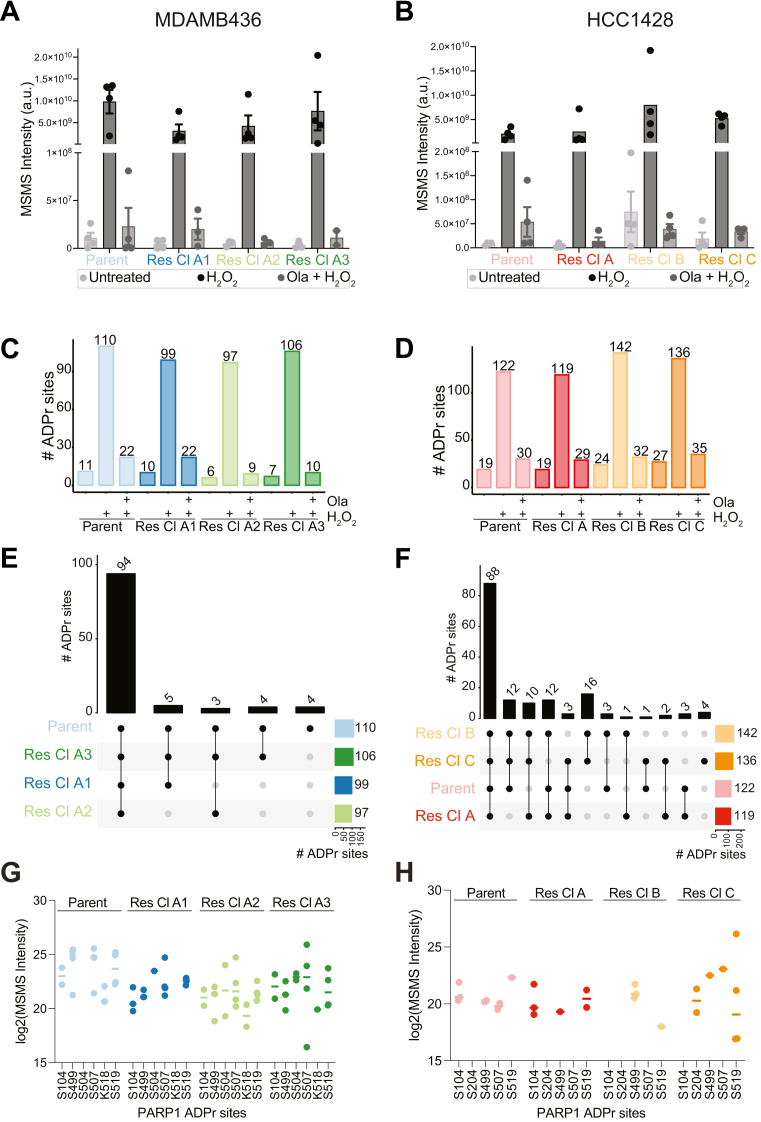
**ADPr response to H_2_O_2_-induced DNA damage is homogeneous in MDAMB436 and HCC1428 parent and OR cells, with modest differences corresponding to protein-level changes.***A-B*, the sum of ADP-ribosylated peptide signal (abundance), in untreated, H_2_O_2_ treated, and Olaparib + H_2_O_2_-treated MDAMB436 (*A*) or HCC1428 (*B*) parent and OR cell lines, identified by MS analysis of ADPr-enriched samples. *C-D*, total number of quantified ADPr sites in untreated, H_2_O_2_-treated, and Olaparib + H_2_O_2_-treated MDAMB436 (*C*) or HCC1428 (*D*) cells. *E-F*, ADPr site overlap across H_2_O_2_-treated MDAMB436 (*E*) or HCC1428 (*F*) cells. *G*-*H*. ADPr intensities of all identified PARP1 sites in H_2_O_2_-treated MDAMB436 (*G*) or HCC1428 (*G*) cells. Lines indicate mean intensity values. n = 4 per condition.

Because proteins in the ADPr pathway were differentially regulated in some or all of the OR cell lines in both MDAMB436 and HCC1428 cells, we investigated how this would affect DNA damage-induced ADPr signaling. We used our previously described Af1521-based enrichment and MS strategy to characterize ADP-ribosylation on a site-level (41, 42, 43) in untreated, H_2_O_2_-treated, and Olaparib (Ola) + H_2_O_2_-treated cells (Fig. 1*H*). As expected, the total ADPr peptide intensity (Fig. 6, *A* and *B*) and number of ADPr sites (Fig. 6, *C* and *D*) were much higher in H_2_O_2_-treated cells compared to untreated and Olaparib-pretreated cells in all cell lines. This is consistent with the findings from the WB analysis (Fig. 1, *F* and *G*). In total, we identified 182 and 219 ADPr sites on 119 and 159 proteins in MDAMB436 and HCC1428 cells, respectively (Supplemental Fig. S3, *A* and *C*). We quantified 110 and 155 ADPr sites in 78 and 120 proteins in total in MDAMB436 and HCC1428 cells, respectively (Supplemental Fig. S3, *A* and *C*). The number of ADPr sites was similar when comparing parent and OR cell lines for MDAMB436 (Fig. 6*C*), whereas a slight increase was seen in the OR clones B and C for HCC1428, corresponding to the clones with the highest upregulation of the PARP1 co-factor HPF1 (Fig. 6*D*). Consistent with previous reports (77, 78), serine was the most abundant ADP-ribosylated amino acid, with around 90% of ADPr sites localizing to serine residues in both cell lines (Supplemental Fig. S3, *B* and *D*). Moreover, the ADPr target proteins identified in both MDAMB436 and HCC1428 cell lines are involved in various DNA damage repair pathways (Supplemental Fig. S3, *E* and *F*).

Additionally, there was a large overlap of all the sites identified in H_2_O_2_-treated parent cells and their respective OR counterparts (Fig. 6, *E* and *F*), suggesting ADPr signaling is homogeneous across Olaparib-sensitive MDAMB436 and HCC1428 parent cells and their resistant counterparts. It was interesting to note that 10 ADPr sites were identified only in the HCC1428 OR cells but not the parent cells, and 16 ADPr sites were uniquely identified in HCC1428 OR cells B and C (Fig. 6*F*). However, these sites were on target proteins that are spread out across the different enriched pathways (Supplemental Fig. S3*F*). A similar homogeneity was observed in untreated and Ola + H_2_O_2_-treated cells (Supplemental Fig. S3, *G*–*J*). Interestingly, seven unique ADPr sites were identified in MDAMB436 OR A3 in Ola + H_2_O_2_-treated conditions (Supplemental Fig. S3*G*), but these were also identified in all H_2_O_2_-treated cells, suggesting that Olaparib may be less effective at inhibiting H_2_O_2_-induced PARP activity in this cell line.

Consistent with our previous studies (62, 77), most of the ADPr signal comes from sites on histones H2BS7, H3S11, and PARP1 in all conditions and cell lines (Supplemental Fig. S3, *K* and *L*). Six and five PARP1 auto-modification sites were identified in the MDAMB436 and HCC1428 cell lines, respectively, but there were no significant differences in PARP1 auto-modification across each parent cell line and its OR counterparts (Fig. 6, *G* and *H*). Together, these results suggest a homogeneous DNA damage ADPr signaling response, even in cells that develop resistance to PARPi, and further underscore that OR in these cell lines is a consequence of protein-level changes rather than remodeling of the ADPr pathway.

### Phosphoproteome analysis Reveals Distinct Phosphorylation-Dependent Signaling Rewiring of DNA Damage Repair Pathways in Olaparib-Resistant MDAMB436, But Not HCC1428 Cells

To understand how phosphorylation signaling contributes to PARPi resistance, we characterized the phosphoproteomes of untreated, H_2_O_2_-treated, and Ola + H_2_O_2_-treated MDAMB436 and HCC1428 parent and OR cells. Phosphorylated peptides were enriched by immobilized metal affinity chromatography (IMAC) using Zirconium-IMAC beads and measured by LC-MS/MS operating in data-independent acquisition (DIA) mode (44, 79, 80). One replicate of the H_2_O_2_-treated MDAMB436 OR Cl A3 sample was lost during MS acquisition and could not be analyzed.

In MDAMB436 cells, 5000 to 6000 phosphorylated sites were identified after filtering for sites quantified in at least three replicates of at least one condition (Supplemental Fig. S4*A*). The most phosphorylation sites were consistently identified in parent cells, and across all cell lines, the most phosphorylation sites were identified in Ola + H_2_O_2_-treated cells. PCA of untreated cells showed that the parent cells clustered separately from the three OR cell lines (Supplemental Fig. S4*B*), consistent with transcriptome and proteome profiles (Fig. 2, *A* and *B*). Within each cell line, PCA showed that Ola + H_2_O_2_-treated cells clustered separately from both untreated and H_2_O_2_-only conditions (Supplemental Fig. S4*C*), suggesting that it was the 1-h pre-treatment with Olaparib that induced large-scale phosphorylation signaling changes rather than H_2_O_2_ treatment. This is different from ADPr, which is specifically induced by H_2_O_2_. Moreover, the presence of thousands of phosphorylation sites in untreated cells suggests that while H_2_O_2_ treatment is a specific ADPr stimulus, it is not the case for phosphorylation. This is unsurprising because signaling pathways involving phosphorylation are far more widespread in human cells (81, 82).

**Fig. 7:**
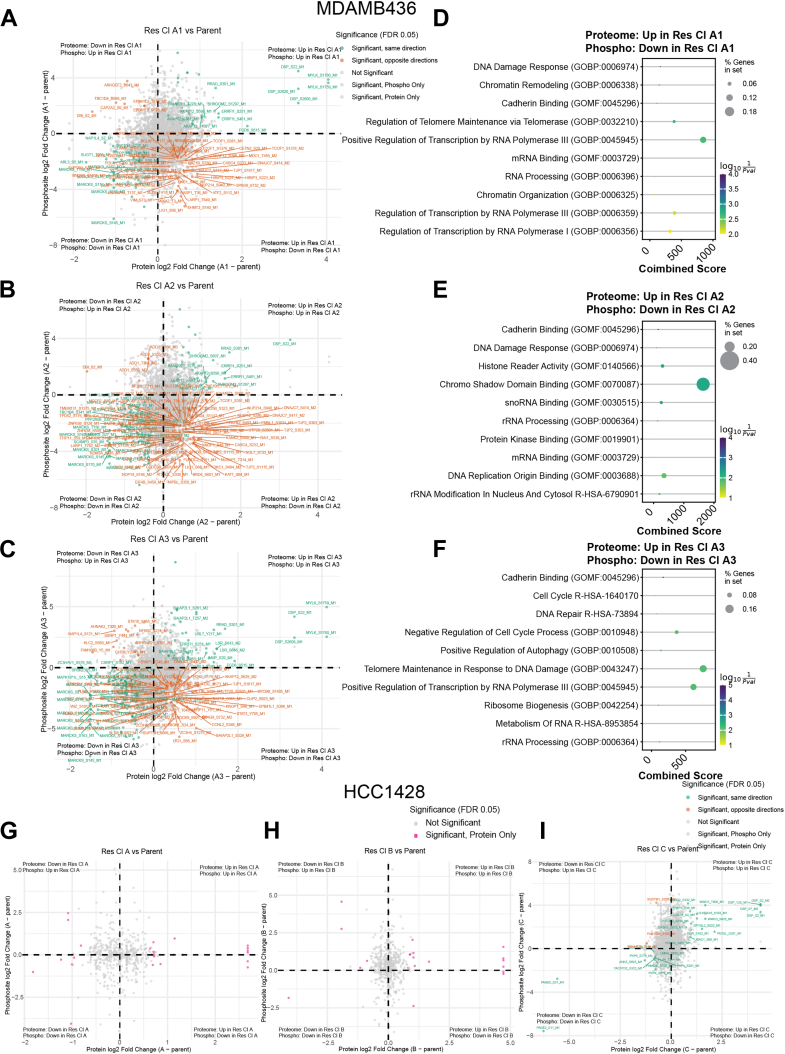
**Phosphorylation-specific regulation of DNA damage response proteins observed in OR MDAMB436 cells.***A–C*, scatter plots show log_2_ fold changes in phosphopeptide abundance (y-axis) *versus* corresponding protein abundance (x-axis) for individual phosphorylation sites in untreated MDAMB436 OR A1 (*A*), OR A2 (*B*), and OR A3 (*C*) compared to MDAMB436 Parent cells. *D–F*, functional enrichment on proteins that are significantly upregulated in OR A1 (*D*), OR A2 (*E*), or OR A3 (*F*) relative to MDAMB436 parent cells, whereas phosphorylation is significantly downregulated, from the bottom right quadrants of their corresponding scatter plots. The dot plots show the top 10 significantly enriched pathways by Gene Ontology (GO) Biological Process (GOBP), GO Molecular Function (GOMF), or Reactome pathway (R-HSA), with dot size representing the percentage of genes in the input set and dots colored by log10 (1/*p*-value). The combined score is the Fisher exact test log *p*-value multiplied by the Z-score of the deviation from the expected rank. Functional annotation was performed using Enrichr in GSEApy. *G–I*, scatter plots show log_2_ fold changes in phosphosite abundance (y-axis) *versus* corresponding protein abundance (x-axis) for individual phosphorylation sites in untreated HCC1428 OR A (*H*), OR B (*H*), and OR C (*I*) compared to HCC1428 Parent cells. In *panels* (*A*), (*B*), (*C*), (*G*), (*H*), and (*I*), phosphosites are color-coded by the direction and significance of changes at the phosphopeptide and protein levels in each OR cell line relative to the Parent cells as follows: Same direction: both phosphosite and protein significantly up- or downregulated; Opposite directions: significant change in opposite directions (phosphopeptide up, protein down and vice versa); Phospho only: significant change at phosphopeptide level only; Protein only: significant change at protein-level only; Not significant: no significant change at either level. Statistical significance is based on 2-sided T tests, adjusted for multiple testing and truncated based on a false discovery rate (FDR) of 0.05.

To evaluate whether phosphorylation differences reflect signaling regulation beyond protein abundance changes, we performed an integrated analysis of phosphopeptide *versus* protein-level changes in untreated cells. For each OR cell line, we compared log_2_ fold changes in phosphopeptide abundance to those of the corresponding protein relative to the parent cell line. In MDAMB436 OR A1, A2, and A3 cells, we found a large subset of proteins that were significantly upregulated at the protein level but showed significant downregulation at specific phosphorylation sites (Fig. 7, *A*–*C*). Functional enrichment of these proteins showed that in all three OR cell lines, these proteins are involved in pathways related to DNA damage response and cell cycle regulation (Fig. 7, *D*–*F*, Supplemental Table 6).

Specifically, proteins such as TOP2A, POLA1, MDC1, UIMC, BOD1L1, and BAZ1B were enriched in the gene ontology (GO) term “DNA Damage Response” in OR A1 and OR A2, while in OR A3, these and additional proteins including CDK2, SMARCA5, and MLH1 were enriched in the “DNA Repair” Reactome pathway. Notably, many of these proteins function at the interface of DNA replication, repair, and cell cycle progression (83, 84, 85, 86, 87, 88), suggesting that phosphorylation of DNA repair regulators is selectively modulated in resistant cells. These findings support the idea that in MDAMB436 cells, regulation of the phosphorylation level plays a role in rewiring genome maintenance and cell cycle signaling pathways during the acquisition of Olaparib resistance.

Because HDR proteins, which were upregulated in OR cells, are targets for phosphorylation, we investigated whether phosphorylation site intensities of HDR proteins were regulated differently across the different treatments. Of the HDR proteins identified in MDAMB436 proteomes (Fig. 2*D*), TOPBP1, ATM, RAD50, and RRAD were identified in our phosphoproteome (Supplemental Fig. S4*D*). Overall, we observed similar phosphorylation patterns across all cell lines – for example, RRAD phosphorylation was lowest in untreated cells and highest in Ola + H_2_O_2_-treated cells, whereas TOPBP1_S888 intensities were highest in untreated cells and lowest in Ola + H_2_O_2_-treated cells. Moreover, within each treatment condition, phosphorylation site intensities were overall higher in OR cell lines compared to parent cells, correlating with increased protein levels.

In HCC1428 cells, we identified around 50% fewer phosphorylation sites compared with MDAMB436 cells (Supplemental Fig. S4*E*). In each cell line, the fewest ADPr sites were identified in Ola + H_2_O_2_-treated cells. PCA of untreated cells showed distinct clustering of each cell line, consistent with transcriptome and proteome PCAs (Supplemental Fig. S4*F*). Within each cell line, untreated, PCA of the three treatment conditions showed that untreated samples were the most distinct in all HCC1428 cell lines (Supplemental Fig. S4*G*).

To further evaluate whether phosphorylation differences in untreated cell lines reflected regulation independent of protein-level changes, we examined phosphopeptide *versus* protein abundance log_2_ fold changes in each HCC1428 OR cell line relative to the parent cells (Fig. 7, *G* and *I*). In OR cell lines A and B, we did not identify any phosphorylation sites that were significantly regulated at both the protein and phosphorylation levels. In OR cell line C, most of such sites were regulated in the same direction (*i.e.*, increased or decreased at both levels), most sites significantly regulated on both the protein and phosphoprotein level were changing in the same direction.

Because phosphorylation was also more widespread than ADP-ribosylation in untreated and Ola + H_2_O_2_-treated HCC1428 cells, we next investigated the differential regulation of phosphorylation in proteins associated with the ‘Signaling by Rho GTPases’ Reactome term, which was significantly enriched in OR Cl A and OR Cl C. Among the Rho GTPase signaling proteins identified in the HCC1428 proteomes (Fig. 5, *F* and *G*), PLEKHG3, ERCC6L, ARHGEF12, and KLC4 were phosphorylated in our study (Supplemental Fig. S4*H*). As observed at the protein levels, phosphorylation was higher in OR cell lines compared to parent cells, and the highest levels were identified in OR Cl C.

Together, these results highlight key differences in the role of phosphorylation between the 2 cell line models. In MDAMB436 cells, we identified a subset of DNA damage response and cell cycle proteins that were differentially regulated at the phosphorylation level independent of changes in protein abundance, suggesting phosphorylation-specific signaling rewiring during the acquisition of Olaparib resistance. In contrast, we found fewer phosphorylation-specific changes in HCC1428 cells overall, and the phosphorylation changes we found largely mirrored protein-level differences, particularly in OR Cl C. These findings indicate that while phosphoproteome signaling changes contribute to resistance in MDAMB436 cells, they may play a more limited or distinct role in HCC1428 cells.

## Discussion

In this study, we profiled and analyzed OR cells generated from two BRCA1/2 mutant breast cancer cell lines at the transcriptome, proteome, ADP-ribosylome, and phosphoproteome levels to investigate the changes they undergo when they acquire Olaparib resistance. We observed that both BRCA1 protein and RNA levels were significantly increased in OR MDAMB436 cells compared to the parent cells. Additionally, we found that other upregulated proteins in OR cell lines are significantly enriched in the “HDR Through Single Strand Annealing” Reactome term. These findings strongly suggest that the OR MDAMB436 cells have regained HDR function. Consistent with increased protein levels, phosphorylation of some of these proteins has also increased. Our findings, therefore, suggest that the reestablishment of BRCA1 expression and HDR function drives Olaparib resistance in MDAMB436 cells. That all three OR cells developed resistance by stabilizing BRCA1, undermining the synthetic lethal interplay between BRCA1 and PARP1, is not surprising and highlights the selection pressure on the cells to restore BRCA1 function as a PARPi resistance strategy. This is a well-described Olaparib resistance development strategy for cancer patients with similar BRCA1 mutations (32, 69).

However, it is still unclear how BRCA1 expression is reestablished in the OR cells. We found that the distribution of nucleotide reads across exonic regions was similar in MDAMB436 parent cells and OR cells, and a variant calling analysis did not reveal mRNA sequence changes in the OR cells that could be contributing to increased stability of BRCA1. We also did not identify peptides corresponding to the BRCT region in the OR cell proteomes. In a similar study where MDAMB436 cells with acquired Rucaparib resistance and Olaparib cross-resistance were characterized, BRCA1 was stabilized by increased binding to heat shock protein 90 (HSP90) (52). It is possible that similar mechanisms are at play in these cell lines, or that BRCA1 is stabilized through increased interactions with other proteins.

We also observed that OR MDAMB436 cells had decreased PARP1 protein levels, and there was a modest reduction in H_2_O_2_-induced ADP-ribosylation and diminished PARP1 automodification upon H_2_O_2_ treatment. It is intriguing to explore whether reduced PARP1 expression in OR cells results from a lower DNA damage burden, linked to the restored HDR function facilitated by BRCA1 stabilization. In an earlier study, we found that ADPr PARP1 levels were highest in untreated MDAMB436 cells compared to the other breast cancer cell lines (30), suggesting increased PARP1-mediated DNA damage repair activity. In this study, we observed significantly lower levels of phosphorylated histone γH2AX at serine 140 in OR cells compared to parent cells in untreated conditions, supporting the idea that basal levels of DNA damage are reduced in OR MDAMB436 cells expressing BRCA1. However, another likely explanation is that because the cells are cultured in the presence of Olaparib, downregulating PARP1 expression could also be a mechanism to reduce the toxic effects of trapped PARP1 on damaged DNA (63).

While resistance to Olaparib in MDAMB436 cells was likely caused by restoration of BRCA1, this was not the case in HCC1428 cells, as all three HCC1428 OR cell lines still expressed BRCA2 containing the deletion originally identified in the parent cells. We therefore investigated other reported changes that cells could undergo when BRCA2 is mutated (69). Considering that the HCC1428 OR cells tolerate only 3 to 6 times more Olaparib compared to parent cells, it is not surprising that the changes in these cells were on a smaller scale.

We found that FANCD2 was significantly upregulated at the RNA and protein level in all OR HCC1428 cell lines. FANCD2 is known to be synthetically lethal with BRCA2 (89). In the absence of functional BRCA2, FANCD2 limits replication fork progression, thereby preventing uncontrolled replication leading to genomic instability (31). There is also an association between BRCA2 loss and increased FANCD2 mRNA levels in breast cancer cell lines and tumors (31, 89). Finally, depletion of FANCD2 was shown to enhance Olaparib sensitivity in BRCA2-deficient cells (31). These observations raise the possibility that upregulation of FANCD2 contributes to the acquisition of Olaparib resistance in HCC1428 cells.

We also found that HPF1 levels were increased in OR HCC1428 cells, and this had modest effects on DNA damage and ADPr signaling. We detected more ADPr sites in OR cell lines B and C, which had the highest increases in HPF1. Because HPF1 is known to bind to PARP1 and direct ADP-ribosylation to serine residues (12), these observations suggest that DNA damage PARP1 activity is increased in HPF1-upregulated OR cells, although PARP1 expression levels are unchanged.

PARP1 uses NAD+ as a cofactor in the ADPr enzymatic reaction, and NAD+ levels in cells drop significantly when DNA damage is activated (73). It is therefore notable that in OR HCC1428 cells, which had increased NAMPT expression, nicotinamide levels were lower while NAD+ and NADH were higher compared to HCC1428 parent cells. Together, these suggest that the metabolism of HCC1428 OR cells adapts to accommodate an increased need for NAD+ consumption due to increased PARP activity during DNA damage signaling.

In a previous study, we showed that PARP1, HPF1, and ARH3 protein levels were higher in MDAMB436 cells compared to HCC1428 cells (30). Interestingly, we also found that NAMPT levels were significantly higher in MDAMB436 cells, which were the most sensitive to the PARPi tested. This suggests that NAD+ biogenesis is upregulated in these cells, probably in response to increased DNA damage, NAD+ consumption by PARPs, and indicates that MDAMB436 parent cells are under a higher basal level of DNA damage stress. Unsurprisingly, NAMPT expression is significantly downregulated in OR MDAMB436 cells. However, nicotinamide levels were significantly lower in all OR cells compared to parent cells. At first glance, this is unexpected given the findings in OR HCC1428 cells. However, considering that BRCA1 is reestablished, PARP1 expression is downregulated, and ɣH2AX is reduced in OR MDAMB436 cells, the OR cells have a reduced need for NAD+, as the DNA damage burden is reduced in these cells.

Functional annotation revealed that Rho GTPase signaling pathway proteins were upregulated in OR HCC1428 cells. Upregulation of Rho signaling in cancer cells contributes to tumor metastasis through increased inhibition of apoptosis, loss of cell polarity, and alteration of cell adhesion (68). We also observed an increase in mRNA levels of NMNAT2, which is associated with higher tumor grade, stage, and metastasis in colorectal cancer (90). Although not directly linked to Olaparib resistance development, it is also interesting to note that the cells have undergone changes associated with cancer progression after prolonged Olaparib exposure.

In both MDAMB436 and HCC1428 cells, we observed that RNA levels did not consistently correlate with protein levels, but this is not surprising due to the various regulatory layers between RNA and the final protein product. Factors such as post-transcriptional regulation, translation efficiency, mRNA degradation rates, protein stability, and PTMs influence RNA and protein levels (91). There is a higher correlation between differentially expressed mRNAs and proteins compared to non-differentially expressed counterparts (92), but other factors may impact protein levels independent of RNA levels. For instance, although BARD1 mRNA levels were unchanged, BARD1 protein levels were significantly upregulated in OR cells with stabilized BRCA1 protein. This emphasizes the importance of BRCA1 binding in stabilizing BARD1 (93). Consequently, BARD1 protein levels are decreased in MDAMB436 parent cells lacking stable BRCA1 (56).

Our comparative analysis of untreated Olaparib-resistant and parental cell lines revealed important differences in phosphorylation regulation between the 2 cell types. In OR MDAMB436 cells, we found many phosphorylation-specific changes that were independent of protein-level changes, particularly among proteins involved in DNA damage response, replication, and cell cycle control. This suggests that phosphorylation-specific signaling rewiring contributes to the acquisition or maintenance of Olaparib resistance in these cells. In contrast, we found fewer phosphorylation-specific differences in OR HCC1428 Olaparib-resistant cell lines, and most phosphorylation changes corresponded to changes in protein abundance. These suggest that phosphorylation signaling may play a more prominent role in acquired resistance in MDAMB436 cells than in HCC1428 cells. One possible explanation is that restoring BRCA1 expression in MDAMB436 cells may not only reconstitute HDR functionality but also lead to modulation of other DNA damage signaling pathways. For example, we observed reduced phosphorylation of histone H2AX in untreated resistant MDAMB436 cells, indicating a lower burden of endogenous DNA damage upon restoration of HDR functionality in these cells re-expressing stable BRCA1. This could decrease the need for constant activation of other DNA damage response pathways, potentially reshaping the phosphorylation landscape as part of a broader cellular adaptation to restored DNA damage repair functionality.

These results underscore the importance of considering both protein abundance and phosphorylation-specific changes when investigating resistance mechanisms. While global oxidative stress induced by H_2_O_2_ (62, 94) may obscure pathway-specific phosphorylation signaling, we were still able to elucidate resistance-associated modifications by analyzing the untreated cells. Future studies using more targeted perturbations—such as inhibition of kinases known to regulate DNA repair, including MAPK1 and MAPK3, which were significantly upregulated in HCC1428-resistant cells—may help clarify whether specific kinase-driven phosphorylation signaling contributes to resistance in a cell line–dependent manner.

Our multi-omics analysis of MDAMB436 and HCC1428 cells with acquired Olaparib resistance is the first study that comprehensively covers transcription, protein expression, ADP-ribosylation, and phosphorylation, providing substantial insight into the cellular changes associated with PARPi resistance. We characterize alterations in PARP1, delineate effects on DNA damage ADPr signaling, and identify other changes in Olaparib-resistant BRCA1/2 mutant breast cancer cells. These changes include reduced DNA damage stress burden and modulation of NAD+ metabolism, which is an intriguing avenue for further investigation. Our study also highlights the complex and seemingly disparate pathways that can change in cells as they acquire resistance to PARP inhibitors.

## Data Availability

The mass spectrometry data have been deposited in the ProteomeXchange Consortium via the PRIDE partner repository (95) with the dataset identifiers PXD060046 for the proteome and phosphoproteome data, and PXD060950 for the ADP-ribosylome data. Annotated spectra for proteome data can be accessed by adding the corresponding raw files into the ‘Proteome_mdamb436_intermediatefiles’ and ‘Proteome_hcc1428_intermediatefiles’ folders for MDAMB436 and HCC1428 cells, respectively, loading the parameters to Maxquant version 2.0.3.1, navigating to the ‘Viewer’ tab, and searching for the protein of interest by its Uniprot ID or gene name. For ADP-ribosylome data, annotated spectra can be accessed similarly by adding the corresponding raw files to the unzipped ADPrmb436intermediatefiles and ADPrhcc1428intermediatefiles folders and loading the parameters into an instance of MaxQuant version 1.5.3.30. Annotated spectra for the phosphoprotoeme data can be accessed by opening the.SNE file provided in the phosphoproteome search folders in an instance of Spectronaut viewer. The RNA sequencing data have been deposited to the SRA repository with the BioProject ID PRJNA1208227. Metabolomics data have been deposited in the EMBL-EBI MetaboLights database (96) with the identifier MTBLS12109.

## Supporting Information

This article contains supporting information.

## Conflict of Interest

The authors declare that they have no conflicts of interest with the contents of this article.

## Supplementary data

Supplemental Table 1 List of proteins identified in this study, volcano plot significant proteins identified in all OR cell lines *versus* the parent cells, and each individual OR cell line *versus* its parent.

Supplemental Table 2 List of differentially expressed genes between all OR cell lines *versus* the parent cells, and each individual OR cell line *versus* its parent, based on RNA sequencing data.

Supplemental Table 3 List of ADP-ribosylation sites identified in this study, and corresponding evidence.txt files from MaxQuant (related to Fig. 6 and Supplemental Fig. S3).

Supplemental Table 4 List of phosphorylation sites identified in this study (related to Fig. 7).

Supplemental Table 5 Concentrations of quantified NAD metabolites measured in this study (Related to Fig. 5 and Supplemental Fig. S2).

Supplemental Table 6 List of phosphorylation sites with log2 fold changes and significance, merged with matched proteins and their log2 fold changes and significance, for each OR cell line compared to the respective parent. For MDAMB436 cells and HCC1428 OR C, enrichment analysis output tables of genes significantly upregulated at the protein level and significantly downregulated at the phosphopeptide level, and/or genes significantly up- or down-regulated exclusively at the phosphopeptide level.
