# Supplementary material for: Multi-omics Characterization of Acquired Olaparib Resistance in BRCA1 and BRCA2 Mutant Breast Cancer Cell Lines
Source: Mol Cell Proteomics. 2025 Jul 14;24(8):101034. doi: 10.1016/j.mcpro.2025.101034 (PMC12355081; doi:10.1016/j.mcpro.2025.101034)
Supplement: Supplementary Figures [file mmc7.pdf]

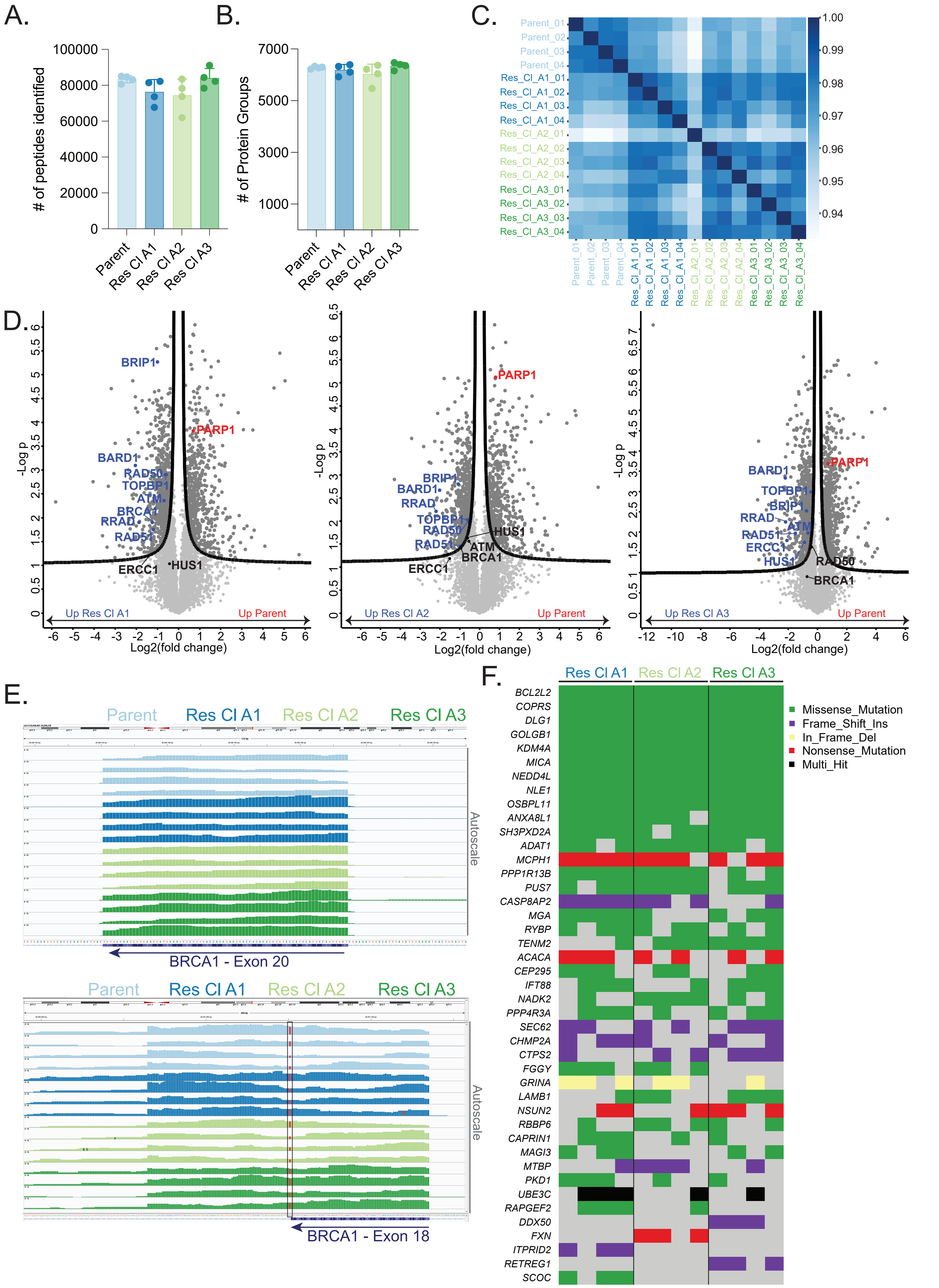

Fig. S1 (main Fig. 2)

Figure S1. Homology-directed repair protein levels, including BARD1, are increased, ɣH2AX is decreased, and PARP1 expression is downregulated in OR MDAMB436 cells with regained BRCA1 expression. A-B. Number of peptides (**A**) and proteins (**B**) identified in MDAMB436 parent and OR cells. n=4 per cell line. **C.** Pearson's correlation coefficient matrix of MDAMB436 parent and OR cells calculated using log2 transformed LFQ intensities of proteins identified in at least three replicates of at least one cell line. **D.** Volcano plots show proteins differentially expressed in each OR cell line compared to parent cells. Each plot shows proteins significantly up (dark gray) in OR cells or parent cells, based on two-sided t-tests with 0.05 FDR. Identified proteins belonging to the 'HDR Through Single Strand Annealing' Reactome term (**Fig. 2F**), are labeled blue if significant or black if not significant. PARP1 is labeled in red. **E.** Integrative Genomics Viewer (IGV) tracks showing mRNA reads of BRCA1 at Exons 20 and 18, with the BRCA1 c.1965+1G>A point mutation in exon 18, identified in all replicates of parent and OR cells, shown in red. The arrows indicate the direction of the open-reading frame. **F.** Oncoplot visualization of the mutations acquired in the MDAMB436 OR cell lines. The variants shown in these plots were selected such as to be detected in minimum 3 out of 4 replicates in at least one of the cell lines, but not detected in the parent cell line.

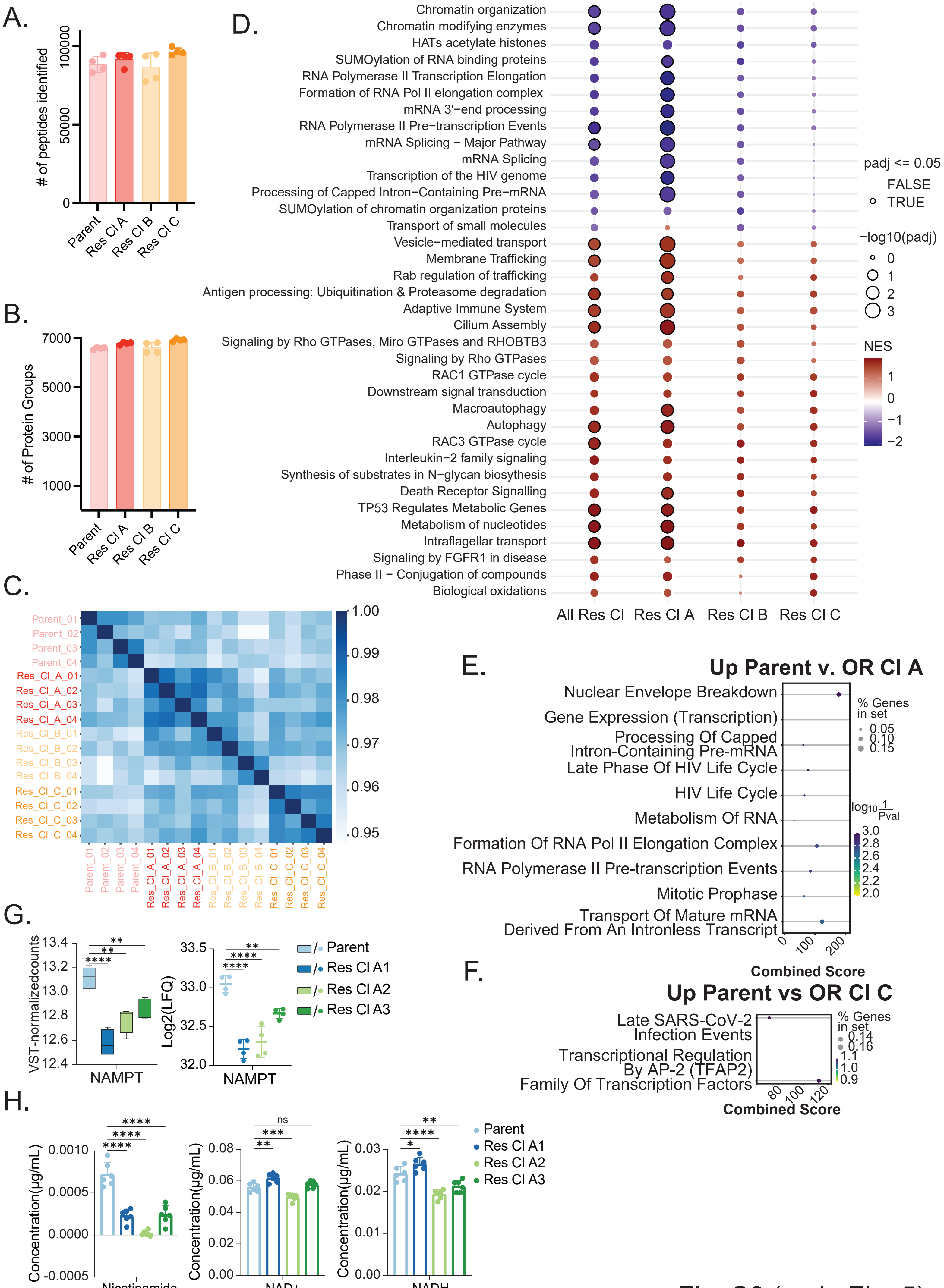

Fig. S2 (main Fig. 5)

Figure S2. Determinants of Olaparib resistance in HCC1428 cells. A-B. Number of peptides (**A**) and proteins (**B**) identified in HCC1428 parent and OR cells. n=4 per cell line. **C.** Pearson's correlation coefficient matrix of HCC1428 parent and OR cells calculated using log2 transformed LFQ intensities of proteins identified in at least three replicates of at least one cell line. **D.** Differential expression analysis and ranked gene set enrichment analysis (GSEA) with Reactome pathways was performed on proteins identified in HCC1428 parent cells and OR cell lines using fGSEA, and the top 15 differentially regulated Reactome pathways in all OR cell lines relative to parent cells and in each OR cell line relative to the parent cells, were plotted on a dotplot. The circle sizes represent the -log10 of the adjusted p-value (padj), and statistical significance (padj <=0.05) is indicated with a black outline. Dots are colored by Normalized Enrichment Score (NES), with positive scores in red and negative scores in blue. **E-F**. Functional enrichment on proteins significantly upregulated in parent relative to OR Cl A (**H**) from volcano plot **Fig. 5C**, or in parent relative to OR Cl C (**I**) from volcano plot **Fig. 5E** as foreground and the human genome as background. The dot plots show the top 10 significantly enriched Reactome pathways, with dot size representing the percentage of genes in the input set, and dots colored by log10 (1/P-value). The combined score is the Fisher exact test log P-value multiplied by the Z-score of the deviation from the expected rank. Functional annotation was performed using Enrichr in GSEApy. **G.** NAMPT mRNA (left) and protein (right) expression MDAMB436 parent and OR cells. **H.** Concentration of Nicotinamide, NAD+, and NADH in MDAMB436 parent and OR cells, normalized to the average protein amount per cell line. N=6 per cell line. In **G** and **H**, statistical significance is based on one-way ANOVA with multiple comparisons of mean variance-stabilizing transformation (VST)-normalized counts (RNA), LFQ intensity values (protein), or metabolite concentrations (**K**) for parent versus each OR cell line. P-values were adjusted using the Dunnett test for multiple testing correction. ns = not significant. * = $p < 0.05$; ** = $p < 0.01$; *** = $p < 0.001$; **** = $p < 0.0001$. Error bars represent the standard deviation from the mean.

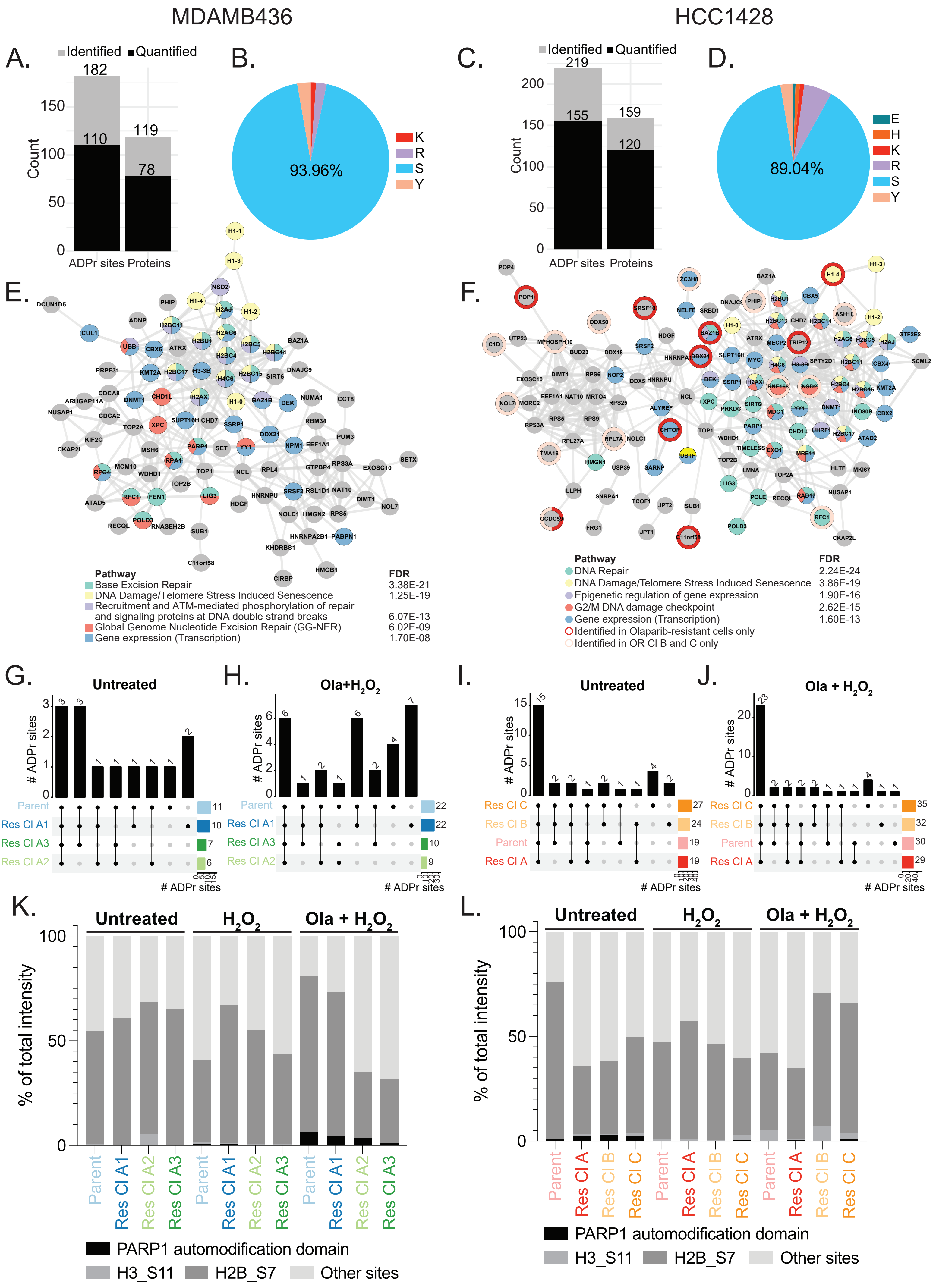

Fig. S3 (main Fig. 6)

Figure S3. ADPr response to H_2O_2-induced DNA damage is homogeneous in MDAMB436 and HCC1428 parent and OR cells, with modest differences corresponding to protein-level changes. **A.** ADPr sites and proteins identified (gray) and quantified (black) in total in MDAMB436 Parent and OR cells. **B.** Amino acid distribution of identified ADPr sites in MDAMB436 Parent and OR cells. **C.** ADPr sites and proteins identified (gray) and quantified (black) in total in HCC1428 Parent and OR cells. **D.** Amino acid distribution of identified ADPr sites in HCC1428 Parent and OR cells. In **A** and **C**, identified sites are ADPr sites with localization score >0.90 and 1% FDR, and quantified sites are identified ADPr sites where intensities were determined in at least 3 replicates of at least one sample. **E-F.** STRING functional association network (Confidence score cutoff = 0.7) shows ADPr target proteins identified in total in MDAMB436 (**E**) or HCC1428 (**F**) parent and OR cell lines. Proteins involved in the top 5 functionally enriched Reactome pathways are colored, and the FDR values of the functional enrichment are indicated. In **F**, a red outer circle indicates ADPr sites identified only in OR cells, and a pink outer circle indicates ADPr sites identified exclusively in OR Cl B and C. Proteins not connected to any others were excluded from the network.**G-H.** ADPr site overlap across the cell lines in untreated (**G**) and Olaparib + H_2O_2-treated (**H**) MDAMB436 cells. **I-J.** ADPr site overlap across the cell lines in untreated (**I**) and Olaparib + H_2O_2-treated (**J**) HCC1428 cells. **K-L.** Intensity distribution of ADPr on the PARP1 automodification domain, Histone H3_S11, Histone H2B_S7, compared to all other identified sites in untreated, H_2O_2-treated, and Olaparib + H_2O_2-treated MDAMB436 (**K**) or HCC1428 (**L**) cells. Intensities of all H2B and H3 subtypes were aggregated by summing them up.

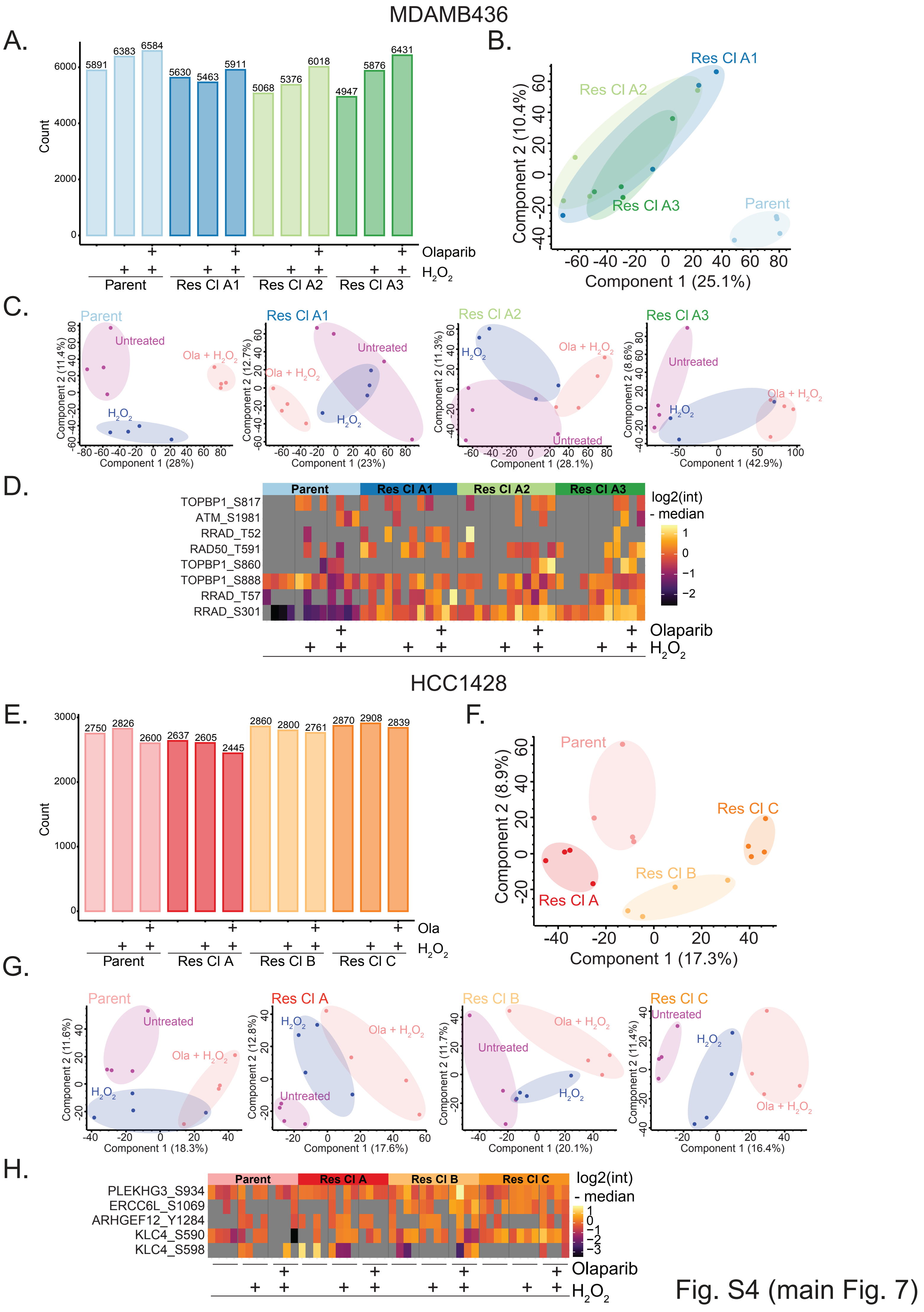

Fig. S4 (main Fig. 7)

Figure S4. Global phosphorylation profiling in MDAMB436 and HCC1428 parent and OR cell lines in Untreated, H_2O_2- or Olaparib + H_2O_2- treated conditions. A. Total count of phosphorylation sites in untreated, H_2O_2-treated, and Olaparib + H_2O_2-treated MDAMB436 parent and OR cells, after filtering for sites quantified in at least 3 out of 4 replicates **B**. PCA plot comparing untreated MDAMB436 parent cells and OR cell lines (A1, A2, and A3) based on MS-derived phosphopeptide intensities. **C**. Four PCA plots showing untreated, H_2O_2-treated, and Olaparib + H_2O_2-treated conditions for each cell line (MDAMB436 parent and OR A1, A2, and A3) based on MS-derived phosphopeptide intensities. **D.** Heatmap of phosphorylation sites for proteins belonging to the 'HDR Through Single Strand Annealing' Reactome term (**Fig. 3B**), which were significantly upregulated in OR cells. **E.** Total count of phosphorylation sites in untreated, H_2O_2-treated, and Olaparib + H_2O_2-treated HCC1428 parent and OR cells, after filtering for sites quantified in at least 3 out of 4 replicates **F**. PCA plot comparing untreated HCC1428 parent cells and OR cell lines (A, B, and C). **G**. Four PCA plots showing untreated, H_2O_2-treated, and Olaparib + H_2O_2-treated conditions for each cell line (HCC1428 parent and OR A, B, and C) based on MS-derived phosphopeptide intensities. **H.** Heatmap of quantified phosphorylation sites for proteins belonging to the 'Signaling by Rho GTPases' Reactome term, which were significantly upregulated in Res Cl A and C (**Fig. 5F & Fig. 5G**).
